# Melatonin Counteracts Mechanical Unloading‐Induced Bone Loss Through YTHDF3‐Mediated m^6^A Modification of *Dapk2* mRNA

**DOI:** 10.1111/jpi.70146

**Published:** 2026-05-18

**Authors:** Quan Sun, Liqun Xu, Zhikui Li, Junfei Zhang, Xiran Zhao, Lijun Zhang, Xiaoyan Zhang, Jiangdong Zhao, Yingjun Tan, Luyao Wang, Ge Zhang, Zebing Hu, Shu Zhang, Fei Shi

**Affiliations:** ^1^ The Key Laboratory of Aerospace Medicine, Ministry of Education The Fourth Military Medical University Xi'an Shaanxi China; ^2^ Department of Gastroenterology and Endocrinology Western Theater Air Force Hospital of PLA Chengdu Sichuan China; ^3^ Department of Otolaryngology Head and Neck Surgery 980th Hospital of PLA Joint Logistics Support Force (Bethune International Peace Hospital) Shijiazhuang Hebei China; ^4^ Department of Otolaryngology Head and Neck Surgery Western Theater Air Force Hospital of PLA Chengdu Sichuan China; ^5^ State Key Laboratory of Space Medicine Fundamentals and Application China Astronaut Research and Training Center Beijing China; ^6^ School of Chinese Medicine, Institute for Advancing Translational Medicine in Bone & Joint Diseases Hong Kong Baptist University Hong Kong SAR China

**Keywords:** DAPK2, mechanical unloading‐induced bone loss, melatonin, *N* ^6^‐methyladenosine, YTHDF3

## Abstract

Disuse osteoporosis, a consequence of prolonged mechanical unloading, is characterized by bone loss and elevated fracture susceptibility. Although melatonin exhibits bone‑anabolic properties, its mechanistic role in the context of mechanical unloading remains elusive. Our findings demonstrate that melatonin promotes osteogenic differentiation and suppresses osteoblast apoptosis, collectively mitigating unloading‑induced osteoporotic bone loss in hindlimb unloading (HLU) mice. Moreover, unloading suppressed YTHDF3 expression in osteoblasts and bone tissue, which was effectively rescued by melatonin administration. Functionally, YTHDF3 potentiated osteoblast differentiation and matrix mineralization while inhibiting apoptotic cell death. At the molecular level, YTHDF3 directly recognized m^6^A‑modified *Dapk2* transcripts and promoted their decay. DAPK2 was characterized as a negative regulator that impedes osteoblast differentiation and survival. Genetic analyses established that melatonin‑driven suppression of DAPK2 and functional recovery of osteoblasts are contingent upon YTHDF3. In summary, we delineate a melatonin/YTHDF3/DAPK2 protective axis that safeguards against unloading‑induced bone deterioration via post‑transcriptional regulation of *Dapk2*, thereby unveiling new mechanistic perspectives and therapeutic opportunities for disuse osteoporosis.

## 1 Introduction

Mechanical unloading serves as a primary etiological driver of disuse osteoporosis, which is frequently observed in individuals experiencing prolonged spaceflight or extended bed rest [1]. This condition manifests clinically through accelerated bone mass reduction, compromised bone microarchitecture, and elevated fracture risk, presenting substantial threats to skeletal integrity in astronauts and chronically immobilized patients [1, 2]. The underlying pathology involves the disruption of skeletal homeostasis due to the absence of mechanical stimulation, characterized by decreased osteoblast‐mediated bone formation coupled with enhanced osteoclast‐driven bone resorption, which collectively drives progressive bone loss [3, 4, 5]. Contemporary therapeutic approaches involving physical rehabilitation and pharmacological interventions, while offering limited clinical benefit, face significant challenges, including insufficient efficacy, notable adverse effects, and poor long‐term treatment adherence [6, 7, 8]. Given the complex pathogenesis of mechanical unloading‐induced osteoporosis—particularly the incompletely elucidated molecular mechanisms governing impaired osteogenesis—systematic investigations of bone metabolic dysregulation under unloading conditions and the identification of pivotal regulatory targets have crucial scientific and clinical importance for advancing pathological understanding and developing novel antiosteoporosis strategies with enhanced therapeutic efficacy and improved safety profiles.

Melatonin, a multifunctional indoleamine hormone primarily secreted by the pineal gland, exhibits potent antioxidant and anti‐inflammatory properties in addition to its well‐established regulatory effect on the circadian rhythm [9, 10, 11]. Particularly relevant to skeletal physiology, melatonin serves as a key regulator of bone metabolism, maintaining the delicate balance between bone formation and resorption while promoting matrix mineralization; thus, melatonin is a promising therapeutic agent for osteoporosis management [12, 13, 14, 15, 16]. While extensive experimental data from both in vitro and in vivo studies have confirmed the osteogenic ability of melatonin, the precise molecular pathways through which it counteracts mechanical unloading‐induced bone loss remain poorly defined. Emerging research indicates that melatonin mediates its biological effects through the regulation of m^6^A RNA modifications. Notably, it alleviates benzene exposure‐induced testicular injury by restoring normal m^6^A methylation patterns [17] and mitigates oncogene‐driven cellular senescence in the ovarian surface epithelium via YTHDF2‐dependent mechanisms [18]. These discoveries reveal novel epigenetic pathways through which melatonin regulates gene expression at the posttranscriptional level. Accumulating evidence suggests that aberrant m^6^A modification is involved in the pathogenesis of skeletal disorders [19, 20, 21, 22]. Nevertheless, the potential interplay between melatonin signaling and m^6^A epitranscriptomic regulation in the context of mechanical unloading‐associated bone metabolic dysregulation remains a completely unexplored research domain worthy of systematic investigation.

As the most abundant RNA modification in eukaryotic systems, m^6^A methylation is dynamically regulated by an integrated network comprising writers (methyltransferases), erasers (demethylases), and readers (recognition proteins) that collectively constitute the m^6^A epitranscriptomic regulatory apparatus [23]. The rapidly expanding family of m^6^A reader proteins demonstrates remarkable functional diversity, with different readers directing distinct RNA processing outcomes following m^6^A recognition. Among these, YTHDF3 stands out as a crucial regulatory component that is capable of direct m^6^A site recognition and functional coordination with YTHDF1 and YTHDF2 to mediate both translational promotion and RNA degradation pathways [24]. While YTHDF3 has been implicated in various pathological conditions, including lung adenocarcinoma, breast cancer, thyroid carcinoma, and hepatic/cardiac fibrotic diseases [25, 26, 27, 28], its role in skeletal biology remains unclear. The literature contains only the seminal report by Feng et al., which established that YTHDF3 enhances *Sox4* mRNA stability to positively regulate osteoblast proliferation and differentiation [29]. Significant knowledge gaps concerning the precise functions of YTHDF3 in disuse‐induced osteopenia, downstream mechanistic pathways, and potential interactions with melatonin signaling remain. Addressing these unanswered questions will not only advance our understanding of the regulatory mechanisms of melatonin in bone metabolism but also reveal innovative therapeutic targets for preventing and treating mechanical unloading‐associated bone loss.

Employing integrated experimental systems comprising osteoblast clinorotation and murine hindlimb unloading (HLU) paradigms, this research revealed that melatonin preserves osteogenic differentiation competence and mineralization functionality, suppresses programmed cell death in osteoblasts, and attenuates mechanical unloading‐mediated bone loss. This pivotal discovery reveals that melatonin reverses the unloading‐induced suppression of the m^6^A reader protein YTHDF3 during osteoporotic progression. Bone‐specific delivery of *Ythdf3* through the use of (DSS)_6_‐liposome nanotechnology partially restored unloading‐compromised osteogenesis. Corroborating these findings, melatonin administration normalized the expression of YTHDF3 in osteoblasts subjected to mechanical unloading. Genetic perturbation approaches demonstrated that *Ythdf3* augmentation enhances osteogenic differentiation while inhibiting apoptosis, whereas *Ythdf3* depletion significantly decreases the pro‐osteogenic, promineralization, and antiapoptotic activities of melatonin, indicating that YTHDF3 is an indispensable molecular mediator of the skeletal protective effects of melatonin under unloading conditions. Mechanistic interrogation through integrated RNA sequencing and RIP‐PCR revealed that YTHDF3 directly interacts with m^6^A‐modified *Dapk2* transcripts, expediting their decay and consequent DAPK2 downregulation. Functional validation confirmed that DAPK2 is a YTHDF3‐regulated effector that constrains osteoblast differentiation and mineralization while promoting apoptotic signaling. Moreover, we demonstrated that melatonin counteracts disuse‐induced osteopenia through modulation of the YTHDF3/DAPK2 signaling cascade. Our investigation provides groundbreaking evidence that melatonin orchestrates osteoblast differentiation, mineralization, apoptosis, and bone anabolic processes under mechanical unloading via the YTHDF3/DAPK2 regulatory circuitry, revealing innovative therapeutic targets for intervention against disuse osteoporosis.

## 2 Materials and Methods

### 2.1 Cell Culture and 2D Clinostat

The MC3T3‐E1 murine preosteoblast cell line was sourced from the Cell Bank of the Chinese Academy of Sciences (Shanghai, China). Cells were cultured in α‐MEM (Gibco, USA) supplemented with 10% fetal bovine serum (Gibco, USA) and 1% penicillin/streptomycin solution (Gibco, USA) at 37°C in a humidified atmosphere containing 5% CO_2_. Experimental groups were treated with 100 nM melatonin (Sigma, USA) for specified durations, with melatonin stock solution prepared in dimethyl sulfoxide (DMSO; Sigma, USA). Luzindole (10 µM) (TargetMol, USA), a competitive melatonin membrane receptor antagonist, was used to test the receptor dependence of melatonin. All experiments utilized cells between passages 4 and 8 and were conducted in triplicate.

Mechanical unloading was simulated using a two‐dimensional (2D) clinostat system (China Astronaut Research and Training Center). MC3T3‐E1 cells were seeded at 5 × 10^5^ cells per flask into specialized 25 cm^2^ clinorotation culture flasks. After cellular adhesion was confirmed, flasks were completely filled with culture medium and securely sealed to ensure bubble‐free conditions. The prepared flasks were then mounted on the clinostat and rotated horizontally at 24 rpm. Static control groups were maintained under identical environmental conditions without rotational stimulation.

### 2.2 Animals and HLU Model

Six‐month‐old male C57BL/6J mice (Animal Center of Air Force Medical University, Xi'an, China) were utilized for HLU model establishment. Animals were randomly allocated to control (CON) and HLU experimental groups. HLU mice were subjected to tail suspension at a 30° inclination using medical‐grade adhesive tape, maintaining consistent mechanical unloading while preserving adequate mobility and unrestricted access to food and water. All animals were housed under strictly controlled environmental conditions (22°C, 12‐h light/dark cycle, 50%–60% humidity). Melatonin‐treated groups received intraperitoneal injections of melatonin (20 mg/kg) every other day, while control groups were administered equivalent volumes of normal saline (NS). For bone‐specific targeted delivery, mice received tail vein injections of either pEX‐*Ythdf3* (2 mg/kg) or negative control pEX plasmid complexed with (AspSerSer)_6_‐liposome (designated as (DSS)_6_‐liposome) on 3 consecutive days prior to unloading initiation. The (DSS)_6_‐liposome‐based targeted delivery methodology was implemented following previously established procedures [30]. After 3 weeks of mechanical unloading, all animals were humanely euthanized, with bilateral femoral and tibial bones collected for subsequent morphological and molecular analyses. Dynamic bone formation parameters were evaluated using dual calcein labeling (Sigma, USA), with fluorochrome injections administered 3 and 10 days preceding terminal sacrifice. All experimental protocols were reviewed and approved by the Institutional Animal Care and Use Committee of Air Force Medical University and conducted in strict adherence to ethical guidelines for laboratory animal welfare.

### 2.3 RNA Extraction and Quantitative Real‐Time PCR (qRT–PCR) Analysis

Total RNA was purified from tissue samples and cultured cells using RNAiso Plus reagent (TaKaRa, Japan) following the manufacturer's specifications. Reverse transcription was performed with PrimeScript RT Master Mix (TaKaRa, Japan) to generate complementary DNA templates. qRT–PCR amplification was conducted using SYBR Premix Ex Taq II on a CFX96 Real‐Time PCR Detection System (Bio‐Rad, USA). Transcript abundance was quantified employing the comparative threshold cycle (2^−ΔΔCt^) method, with normalization to GAPDH expression as an internal control. All primer sequences designed and validated for this investigation are comprehensively detailed in Table S1.

### 2.4 Protein Extraction and Western Blot Analysis

Cellular and tissue protein extracts were prepared utilizing M‐PER Mammalian Protein Extraction Reagent (Thermo Fisher Scientific, USA) supplemented with complete protease and phosphatase inhibitors (Roche, Switzerland). Protein quantification was performed using the Pierce BCA assay (Thermo Fisher Scientific, USA) according to manufacturer's specifications. Equal protein aliquots were separated by 10% SDS‐PAGE and electrophoretically transferred to PVDF membranes (Millipore, USA). Membrane blocking was conducted with 5% nonfat dry milk in TBST. Primary antibody incubations were performed overnight at 4°C using the following specific antibodies: GAPDH (1:50 000; Proteintech, China), YTHDF3 (1:2000; Proteintech, China), DAPK2 (1:2000; Proteintech, China), COL1A1 (1:1000; Abclonal, China), RUNX2 (1:1000; Cell Signaling Technology, USA), OCN (1:1000; Abcam, UK), Bax (1:1000; Cell Signaling Technology, USA), Bcl‐2 (1:1000; Cell Signaling Technology, USA), Cleaved Caspase‐3 (1:1000; Cell Signaling Technology, USA), melatonin receptor 1A (MT1) (1:1,000; Abclonal, China), melatonin receptor 1B (MT2) (1:2000; Bioss, China), and Lamin B (1:10 000; Proteintech, China). Subsequent to primary antibody incubation, membranes were probed with HRP‐conjugated secondary antibody (1:5000; ZSGB‐BIO, China) for 2 h at room temperature. Protein detection was achieved through enhanced chemiluminescence substrate development using a BioRad imaging system. GAPDH and Lamin B were implemented as internal reference controls for quantitative normalization. Densitometric analysis of immunoreactive bands was conducted using ImageJ software.

### 2.5 Cell Transfection

Cells were cultured in 6‐well plates and transfected following established manufacturer's protocols. For targeted gene knockdown, transfection was performed with 80 nM of specific small interfering RNAs (si‐*Ythdf3*, si‐*Dapk2*, si‐*MT1*, and si‐*MT2*) or si‐NC (Genema, China) using Lipofectamine 2000 transfection reagent (Thermo Fisher Scientific, USA) with a 48‐h incubation period. For gain‐of‐function experiments, cells were transfected with 500 ng/μL of either pEX‐*Ythdf3*, pEX‐*Dapk2*, or corresponding empty vector control (pEX; Genema, China) utilizing Lipofectamine RNAiMAX transfection reagent (Thermo Fisher Scientific, USA). Complete siRNA sequences designed and implemented in this study are comprehensively detailed in Table S2.

### 2.6 Alkaline Phosphatase Staining

MC3T3‐E1 cells were cultured in 6‐well plates under osteogenic induction conditions for 7 days, with medium refreshed every second day. Subsequently, cells were fixed with 4% paraformaldehyde for 15 min at room temperature. Alkaline phosphatase (ALP) activity was detected using a BCIP/NBT‐based Alkaline Phosphatase Color Development Kit (Beyotime, China) according to the manufacturer's specifications. After development, cells were rinsed thoroughly, and photomicrographs were obtained using a digital imaging system for documentation and analysis.

### 2.7 Alizarin Red S Staining

MC3T3‐E1 cells were cultured in 12‐well plates under osteogenic induction conditions for 21 days to promote matrix mineralization. Cells were then fixed with ice‐cold 70% ethanol at 4°C for 1 h. After three washes with ddH_2_O, calcium deposition was visualized by staining with 1% Alizarin Red S (ARS) solution (Sigma, USA) for 15 min at room temperature. To reduce nonspecific staining, cells were treated with DPBS for 15 min and washed thoroughly with ddH_2_O. Mineralized nodules were photographed using a digital imaging system for qualitative analysis.

### 2.8 TUNEL Assay

Cellular apoptosis in femoral tissue sections was evaluated through terminal deoxynucleotidyl transferase dUTP nick‐end labeling (TUNEL) methodology utilizing the DeadEnd Fluorometric TUNEL System (Promega, USA). The proportion of TUNEL‐positive nuclei relative to total nuclei was determined using Olympus cellSens Standard imaging software. Quantitative apoptosis data are presented in accordance with the standardized statistical reporting framework delineated by Weissgerber et al., ensuring methodological transparency and reproducibility [31].

### 2.9 Flow Cytometric Analysis of Apoptosis

MC3T3‐E1 cells were harvested by trypsinization (0.125% trypsin), rinsed with phosphate‐buffered saline (PBS), and sedimented by centrifugation at 1000 rpm for 5 min. Programmed cell death was evaluated employing a commercial Annexin V‐FITC/PI Apoptosis Detection Kit (BioVision, USA) following the manufacturer's established protocol. Fluorescently labeled cells were subjected to analysis using a flow cytometer (BD Biosciences, USA), with apoptotic rates quantified through systematic enumeration of Annexin V‐positive cellular subpopulations, distinguishing early apoptotic (Annexin V+/PI−) and late apoptotic/necrotic (Annexin V+/PI+) cells.

### 2.10 Hoechst Staining

Nuclear chromatin morphology was assessed employing the Hoechst 33258 Staining Kit (Beyotime Biotechnology, China) according to the manufacturer's specifications. MC3T3‐E1 cells were fixed with 4% paraformaldehyde and stained with Hoechst 33258 solution to detect characteristic apoptotic nuclear alterations, including chromatin condensation and nuclear fragmentation. High‐resolution fluorescence images were captured using a laser scanning confocal microscope (Carl Zeiss, Germany) configured with optimized excitation/emission filter sets to facilitate precise quantification of apoptotic nuclei through morphological analysis.

### 2.11 Enzyme‐Linked Immunosorbent Assay (ELISA)

Plasma samples were obtained through centrifugation of whole blood at 3000 rpm for 15 min at 4°C. The clarified supernatant was systematically aliquoted into presterilized centrifuge tubes for analytical processing. Circulating melatonin concentrations were determined using a validated enzyme‐linked immunosorbent assay (ELISA) methodology performed according to the manufacturer's standardized protocol. Spectrophotometric absorbance readings at 450 nm primary wavelength were obtained utilizing a microplate spectrophotometer (Omega Bio‐Tek, Germany) with integrated reference wavelength compensation to ensure measurement accuracy.

### 2.12 Immunofluorescence

MC3T3‐E1 cells were plated on glass coverslips in 6‐well plates at an initial density of 1 × 10^5^ cells per well. At 80% confluency, cells were fixed with 4% paraformaldehyde and permeabilized with 0.5% Triton X‐100 for 15 min at room temperature. Nonspecific binding sites were blocked with 5% goat serum for 30 min. Immunofluorescence staining was conducted by incubation with anti‐YTHDF3 primary antibody (1:200 dilution; Proteintech, China) overnight at 4°C, followed by treatment with FITC‐conjugated secondary antibody (1:1000 dilution; Proteintech, China) for 1 h at 37°C. Nuclear counterstaining was performed using DAPI for 10 min under light‐protected conditions. Fluorescence images were captured with a laser scanning confocal microscope (Carl Zeiss, Germany) using appropriate filter configurations for each fluorophore.

### 2.13 Micro‐CT

Femoral samples were fixed in 4% paraformaldehyde and subjected to high‐resolution micro‐computed tomography scanning using an advanced imaging system (Perkin Elmer, USA). A standardized cubic region of interest (ROI) measuring 2.5 × 2.5 × 3 mm^3^ was selected commencing 1.5 mm proximal to the growth plate to ensure consistent evaluation of secondary spongiosa trabecular bone architecture. Three‐dimensional volumetric reconstructions were performed, and key morphometric parameters—including bone mineral density (BMD), bone volume fraction (BV/TV), trabecular number (Tb.N), and trabecular separation (Tb.Sp)—were quantitatively analyzed following internationally recognized guidelines for bone histomorphometric assessment.

### 2.14 Histological Analysis

Post‐Micro‐CT evaluation, femoral specimens underwent thorough decalcification in 10% EDTA solution for 21 days, followed by paraffin embedding and preparation of 5‐μm thick longitudinal sections. Bone tissue sections were processed for Goldner's trichrome staining to quantitatively assess osteoid mineralization status and subjected to immunohistochemical (IHC) localization using validated primary antibodies: rabbit anti‐YTHDF3 (1:2000; Proteintech, China) and rabbit anti‐DAPK2 (1:2000; Proteintech, China). Consecutive sections from major organs including cardiac, hepatic, splenic, pulmonary, and renal tissues were stained with hematoxylin and eosin (H&E) for comprehensive histopathological evaluation. Dynamic bone formation kinetics were assessed via dual fluorochrome labeling with calcein green (Sigma, USA), administered intraperitoneally at 3‐day and 10‐day intervals preceding sacrifice. Tibial specimens were fixed in 4% paraformaldehyde, systematically dehydrated through an ascending ethanol gradient, and embedded in polymethyl methacrylate resin. Undecalcified ground sections were examined by laser scanning confocal microscopy (Carl Zeiss, Germany), and the mineral apposition rate (MAR) was precisely quantified by measuring mean interlabel distances between consecutive calcein deposition fronts using standardized stereological methodologies.

### 2.15 Biomechanical Testing

The biomechanical integrity of femoral bones was evaluated using three‐point bending methodology implemented on an electromechanical testing system (Bose, USA). Specimens were oriented horizontally on two supporting fixtures with an 8 mm span distance, and compressive load was applied vertically at the mid‐diaphyseal location using a constant crosshead displacement rate of 0.02 mm/s until complete structural failure. Postfracture morphological characterization included precise measurement of anteroposterior and mediolateral diameters, as well as cortical thickness, utilizing digital calipers with 0.01 mm metrological resolution. Critical biomechanical parameters—maximum load, structural stiffness, and elastic modulus—were computationally derived from the load‐deformation curves through application of standard Euler–Bernoulli beam theory equations, ensuring accurate quantification of bone mechanical competence.

### 2.16 Nuclear–Cytoplasmic Fractionation

Cellular compartmentalization was achieved using a standardized Nuclear and Cytoplasmic Protein Extraction Kit (Beyotime Biotechnology, China) implemented according to the manufacturer's specifications. Cellular pellets obtained by centrifugation were treated with Cytoplasmic Extraction Reagent A enhanced with comprehensive protease inhibition (1 mM PMSF). After 15‐min incubation at 4°C, Cytoplasmic Extraction Reagent B was introduced to complete cytoplasmic extraction, followed by separation of the cytoplasmic fraction via centrifugation at 12 000*g* for 5 min at 4°C. The nuclear pellet was subsequently reconstituted in Nuclear Extraction Reagent and subjected to controlled ultrasonication on ice to ensure complete nuclear membrane disruption. The nuclear protein extract was isolated by centrifugation at 12 000*g* for 10 min at 4°C. All subcellular fractions were cryopreserved at −80°C under optimized conditions to maintain protein integrity for subsequent proteomic analyses.

### 2.17 RNA Stability Assays

To evaluate transcript stability dynamics, MC3T3‐E1 cells were treated with the RNA polymerase II inhibitor actinomycin D (ActD; 5 μg/mL; MedChemExpress, USA) for predetermined intervals (0, 2, 4, and 6 h) to halt de novo RNA synthesis. Total RNA was isolated utilizing Trizol reagent (Invitrogen, USA) following the manufacturer's standardized protocol. Relative mRNA abundance was precisely quantified by qRT–PCR, and transcript degradation rates were mathematically derived from the exponential decay curves of time‐dependent reduction in mRNA abundance, enabling calculation of mRNA half‐life values.

### 2.18 RNA Immunoprecipitation (RIP)

RNA–protein interactions were investigated using the Magna RIP RNA‐Binding Protein Immunoprecipitation Kit (Millipore, USA) implemented according to the manufacturer's specifications. MC3T3‐E1 cellular extracts were prepared in complete RIP lysis buffer containing comprehensive protease and RNase inhibitor cocktails. Magnetic beads were preconjugated with 5 μg of either specific anti‐YTHDF3 antibody (Proteintech, China) or isotype control mouse IgG (Millipore, USA) through 30‐min affinity coupling at ambient temperature. The immunoconjugated beads were subsequently incubated with clarified cell lysates for 12 h at 4°C under constant gentle agitation. After rigorous washing under high‐stringency conditions, ribonucleoprotein complexes were eluted and subjected to proteinase K digestion. Immunoprecipitated RNA was purified, reverse transcribed, and quantitatively analyzed by SYBR Green‐based real‐time PCR employing sequence‐specific primers targeting *Dapk2* transcripts. YTHDF3 binding enrichment was calculated relative to the nonspecific IgG control using the comparative threshold cycle (2^−ΔΔCt^) method, establishing specific RNA‐protein interaction profiles.

### 2.19 TRAP Staining

TRAP staining was performed to evaluate osteoclast activity. Mouse femurs were fixed in 4% PFA, decalcified in 10% EDTA (pH 7.4) for 2–3 weeks, and embedded in paraffin. Sections (5 μm) were stained with a TRAP staining kit (Sigma‐Aldrich, USA) following the manufacturer's protocol. TRAP‐positive multinucleated cells (≥ 3 nuclei) were counted as osteoclasts in the distal femoral metaphysis.

### 2.20 Dual‐Luciferase Reporter Assay

To determine the specific m^6^A site within DAPK2 regulated by YTHDF3, a dual‐luciferase reporter assay was performed. Wild‐type (WT) DAPK2 sequences containing putative m^6^A sites were amplified and inserted into pGL3 vectors. The corresponding mutant (MUT) sequences, in which the potential *N* ^6^‐methylated adenosine sites were replaced with thymine, were also cloned into pGL3‐Basic vectors (all constructs were generated by Sangon Biotech, Shanghai, China). 293 T cells were co‐transfected with either the WT or MUT reporter plasmid, along with either an empty vector or a YTHDF3 overexpression vector, using a standard transfection reagent. Luciferase activity was measured 48 h post‐transfection using the dual‐luciferase reporter assay system (Sangon Biotech).

### 2.21 Statistical Analysis

All statistical computations were performed using SPSS version 22.0 (IBM Corporation, USA). Continuous quantitative data are expressed as the mean ± SD. All in vitro experiments were performed with three biologically independent replicates. For comparisons between two independent experimental groups, two‐tailed unpaired Student's *t*‐tests were used. Multigroup comparative analyses were conducted using one‐way ANOVA with Fisher's LSD post hoc test to identify specific intergroup differences. Data visualization and graphical representations were created using GraphPad Prism version 9.0 (GraphPad Software Inc., USA). Statistical significance was defined as *p* < 0.05 for all hypothesis testing procedures.

## 3 Results

### 3.1 Melatonin Ameliorates Unloading‐Induced Osteoblast Dysfunction

Building upon established evidence of the osteogenic properties of melatonin, we systematically characterized its temporal and dose‐responsive effects on MC3T3‐E1 cells. A time course analysis revealed that 48 h of melatonin exposure markedly increased the transcript levels of pivotal osteogenic markers (*Alp*, *Col1a1*, *Ocn*, and *Runx2*), with concordant increases in the protein levels of COL1A1, RUNX2, and OCN (Figure S1A,B), indicating enhanced osteogenic differentiation capacity. Through concentration‐gradient screening (10 nM to 100 μM), we identified 100 nM as the most effective concentration, resulting in maximal upregulation of osteogenic transcripts and corresponding protein expression (Figure S1C,D). Consistent osteogenic stimulation was verified by ALP staining (Figure S1E), with concomitant augmentation of mineralized matrix formation evidenced by ARS staining (Figure S1F). These comprehensive analyses established 100 nM melatonin administered for 48 h as the standardized condition for subsequent mechanistic investigations.

**Figure 1:**
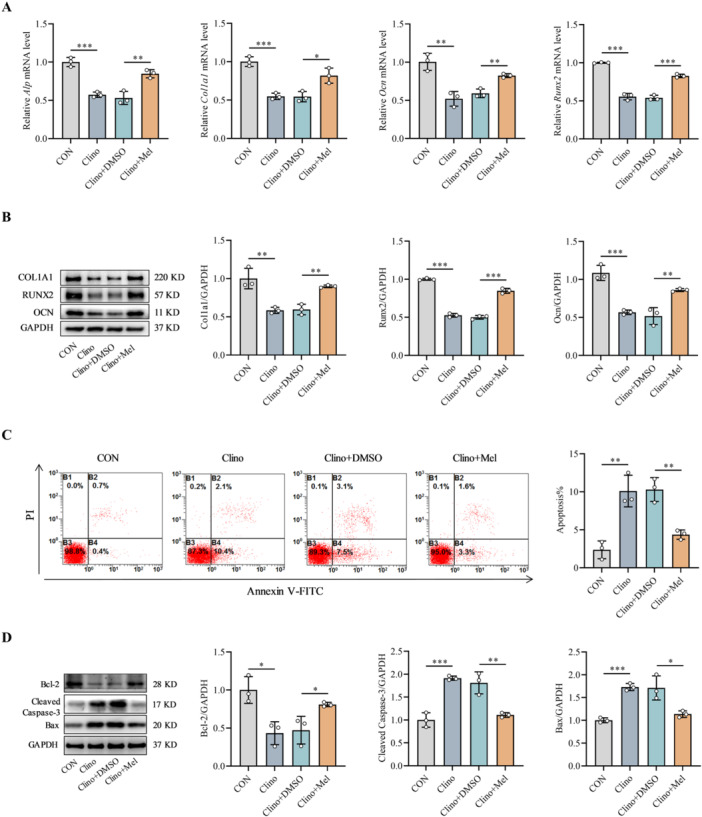
Melatonin ameliorates unloading‐induced osteoblast dysfunction. MC3T3‐E1 cells were cultured in osteogenic induction medium with or without melatonin and subjected to 2D clinorotation to simulate mechanical unloading. (A) qRT–PCR analysis of the mRNA expression of *Alp*, *Col1a1*, *Ocn*, and *Runx2* (*n* = 3). (B) Western blot analysis of the osteogenic marker proteins COL1A1, OCN, and RUNX2 (*n* = 3). (C) Apoptosis analysis by flow cytometry (*n* = 3). (D) Western blot analysis of the apoptosis‐related proteins Bax, cleaved caspase‐3, and Bcl‐2 (*n* = 3). Mel, melatonin. *n* = 3 represents three independent biological replicates. *p < 0.05, **p < 0.01, ***p < 0.001 versus control.

To elucidate the cytoprotective properties of melatonin, we established an in vitro model of mechanical unloading using a 2D clinostat platform. Compared with static control cells (CON), MC3T3‐E1 cells subjected to 48 h of clinorotation exhibited substantial suppression of osteogenic differentiation capacity, as evidenced by significant downregulation of the expression of key osteogenic markers (ALP, COL1A1, OCN, and RUNX2) at both the transcriptional and translational levels (Figure 1A,B). Notably, melatonin supplementation (Clino + Mel) effectively reversed this unloading‐induced suppression, significantly restoring osteogenic marker expression relative to that in the vehicle‐treated unloading group (Clino + DMSO) (Figure 1A,B). In addition to its effects on differentiation, mechanical unloading profoundly promoted apoptosis, as quantified by flow cytometric analysis (Figure 1C). Melatonin administration substantially attenuated this unloading‐induced apoptosis (Figure 1C). At the molecular level, Western blot analysis confirmed that clinorotation upregulated the expression of proapoptotic effectors (Bax and cleaved caspase‐3) while downregulating the expression of the antiapoptotic protein Bcl‐2 (Figure 1D). Melatonin treatment reestablished the equilibrium of these apoptotic regulators, shifting their expression toward physiological levels (Figure 1D). These comprehensive analyses revealed that melatonin preserved osteoblast function under mechanical unloading conditions by concurrently promoting osteogenic differentiation programs and suppressing apoptotic signaling pathways.

### 3.2 Melatonin Preserves Bone Mass, Structure, and Strength in HLU Mice

**Figure 2:**
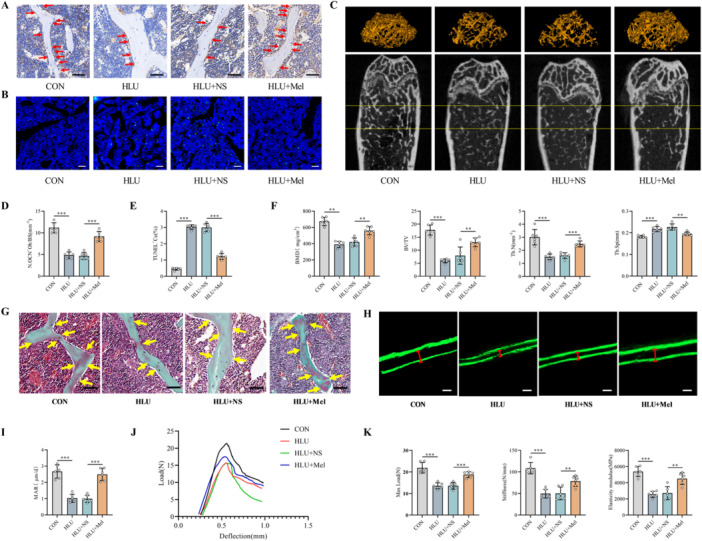
Melatonin preserves bone mass, structure, and strength in HLU mice. (A) Representative images of IHC staining for OCN in the distal femur. Scale bar, 50 μm. (B) Representative images of TUNEL staining of the distal femur. Scale bar, 50 μm. (C) Representative 3D micro‐CT reconstruction images of the ROI in the distal femur. (D) Quantitative analysis of OCN‐positive osteoblast numbers (*n* = 6). (E) Quantitative analysis of TUNEL‐positive cell percentages (*n* = 6). (F) Quantitative analysis of bone parameters, including the bone mineral density (BMD), bone volume/total volume (BV/TV), trabecular number (Tb.N), and trabecular separation (Tb.Sp) (*n* = 6). (G) Goldner's trichrome staining of the distal femur, distinguishing osteoid (red) from mineralized bone (green) for assessing new bone formation (*n* = 6). Scale bar, 50 μm. (H) Representative images of calcein double‐labeling for dynamic histomorphometric analysis of bone formation. Scale bar, 20 μm. (I) Quantitative analysis of the mineral apposition rate (MAR) (*n* = 6). (J) Representative load–displacement curves obtained from three‐point bending tests. (K) Quantitative analysis of biomechanical parameters, including stiffness, maximum load, and elastic modulus (*n* = 6). BS, bone surface; NS, normal saline; Ob, osteoblast; ROI, region of interest. **p < 0.01, ***p < 0.001 versus control.

To systematically evaluate the osteoprotective properties of melatonin, we established an HLU model in C57BL/6J mice subjected to 21 days of tail suspension. Therapeutic melatonin concentrations were achieved through intraperitoneal injection, as verified by significantly elevated serum melatonin levels (Figure S2A). Comprehensive histopathological assessment of major organ systems revealed no evidence of treatment‐related toxicity (Figure S2B). Histomorphometric analysis demonstrated that compared with ambulatory control treatment (CON), mechanical unloading significantly compromised osteoblast function, reduced the number of OCN‐positive osteoblasts and increased the number of TUNEL‐positive apoptotic cells (Figure 2A,B,D,E). Melatonin intervention (HLU + Mel) effectively counteracted these alterations, restoring the osteoblast population and suppressing apoptosis compared with those in normal saline‐treated HLU mice (HLU + NS) (Figure 2A,B,D,E). Micro‐CT evaluation revealed that HLU induced profound trabecular deterioration characterized by sparse trabeculae with structural discontinuities (Figure 2C). Compared with HLU + NS treatment, melatonin treatment substantially preserved trabecular network integrity and BMD (Figure 2C). Quantitative morphometric analysis confirmed that HLU significantly impaired bone mass and microarchitecture, reducing the BMD, BV/TV, and Tb.N while increasing the Tb.Sp (Figure 2F). These deleterious effects were robustly attenuated by melatonin, which resulted in significant improvements in all the measured parameters toward physiological levels (Figure 2F). Moreover, TRAP staining revealed abundant quantities of TRAP‐positive osteoclasts in HLU mice and markedly reduced amounts in melatonin‐treated mice (Figure S2C). Collectively, these findings demonstrate that melatonin counteracts mechanical unloading‐induced osteopenia by sustaining osteoblast viability, reducing apoptosis, suppressing osteoclast activity, and preserving bone mass and microstructural integrity.

To evaluate the effect of melatonin on new bone formation, we performed Goldner's trichrome staining and calcein double‐labeling analysis. Goldner's staining revealed a significant reduction in new bone (green: mineralized bone; red: osteoid) in the HLU group compared with the CON group (Figure 2G). In contrast, compared with HLU + NS treatment, melatonin treatment (HLU + Mel) significantly increased new bone formation (Figure 2G). Concurrently, calcein double‐labeling revealed that mechanical unloading markedly decreased the MAR, whereas melatonin significantly restored the rate of new bone formation (Figure 2H,I). These data collectively indicate that melatonin effectively stimulates osteogenesis in HLU mice. Furthermore, bone loss induced by mechanical unloading substantially increases fracture risk, largely because of the deterioration of bone biomechanical properties. To evaluate whether melatonin counteracts this effect, we performed three‐point bending tests. The load–displacement curves demonstrated that HLU significantly compromised the biomechanical strength of bone, whereas exogenous melatonin supplementation markedly improved the mechanical performance of bone (Figure 2J). Consistent with these findings, specific biomechanical parameters—including stiffness, maximum load, and elastic modulus—were significantly reduced in HLU mice, and these reductions were substantially reversed by melatonin treatment (Figure 2K). Together, these results demonstrate that melatonin effectively restores bone formation and bone biomechanical competence in HLU mice.

### 3.3 Melatonin Effectively Restores the Expression of the Mechanosensitive m^6^A Reader YTHDF3 Through MT2 Receptor Under Mechanical Unloading Conditions

To elucidate the role of m^6^A reader proteins in the protective effect of melatonin against mechanical unloading‐induced bone loss and to identify pivotal regulators, we conducted a systematic screening. In MC3T3‐E1 cells, 48 h of melatonin treatment induced the most substantial upregulation of *Ythdf3* mRNA among all the reader proteins (Figure S3A). In contrast, 48 h of clinorotation resulted in the most pronounced downregulation of *Ythdf3* (Figure S3B), directing our focus to YTHDF3. Time‐course analysis confirmed that both YTHDF3 mRNA and protein levels were significantly suppressed after 24–72 h of clinorotation (Figure S3C,D). We next examined the ability of melatonin to restore YTHDF3 expression under unloading conditions. In cells exposed to 48 h of clinorotation, melatonin treatment significantly reversed the suppression of both *Ythdf3* mRNA (Figure S3E) and YTHDF3 protein expression (Figure S3F). Corroborating these in vitro observations, HLU mice reduced YTHDF3 expression in femoral bone tissue, an effect that was substantially reversed by melatonin treatment at both the transcriptional and translational levels (Figure S3G,H). IHC staining further confirmed this restoration (Figure S3I,J).

However, whether melatonin acts through its classical receptors to exert these effects remains unknown. Luzindole, a competitive melatonin membrane receptor antagonist, was used to address this question. Treatment of osteoblasts with Luzindole (10 µM) markedly inhibited melatonin‐induced upregulation of YTHDF3 (Figure S4A,B). Moreover, Luzindole also counteracted the protective effects of melatonin against the suppression of YTHDF3 expression induced by 2D clinorotation (Figure S4C,D). To further determine whether melatonin signals through MT1 or MT2 receptors, MC3T3‐E1 cells were transfected with specific siRNAs targeting MT1 or MT2. Two independent siRNAs effectively knocked down each receptor (Figure S4E–H). Under normal gravity, melatonin promoted YTHDF3 expression. Knockdown of MT1 did not significantly alter these effects, whereas MT2 knockdown markedly attenuated them (Figure S1). These findings were further validated under 2D clinorotation conditions, where only MT2 knockdown inhibited melatonin's rescue of YTHDF3 expression in osteoblasts subjected to mechanical unloading (Figure S4K,L). Collectively, these results demonstrate that melatonin counteracts mechanical unloading‐induced suppression of the mechanosensitive m^6^A reader YTHDF3 in osteoblasts specifically through the MT2 receptor.

### 3.4 YTHDF3 Orchestrates Osteogenic Progression by Enhancing Differentiation and Mineralization While Simultaneously Inhibiting Apoptosis

On the basis of the observed effects of melatonin on promoting osteoblast differentiation, inhibiting apoptosis, and upregulating YTHDF3 expression under mechanical unloading, we proceeded to elucidate the specific role of YTHDF3 in osteogenic differentiation, mineralization, and apoptosis. MC3T3‐E1 cells were induced to differentiate in osteogenic medium and harvested at designated time points (Days 0, 2, 4, 7, 10, and 14) for the analysis of YTHDF3 and osteogenic marker expression. The qRT–PCR results revealed a progressive increase in the mRNA levels of the osteogenic markers *Col1a1*, *Ocn*, and *Runx2* during differentiation, which was paralleled by upregulation of the *Ythdf3* transcript (Figure S5A). Western blot analysis confirmed a corresponding increase in expression at the protein level (Figure S5B), suggesting that YTHDF3 is involved in osteoblast differentiation.

**Figure 3:**
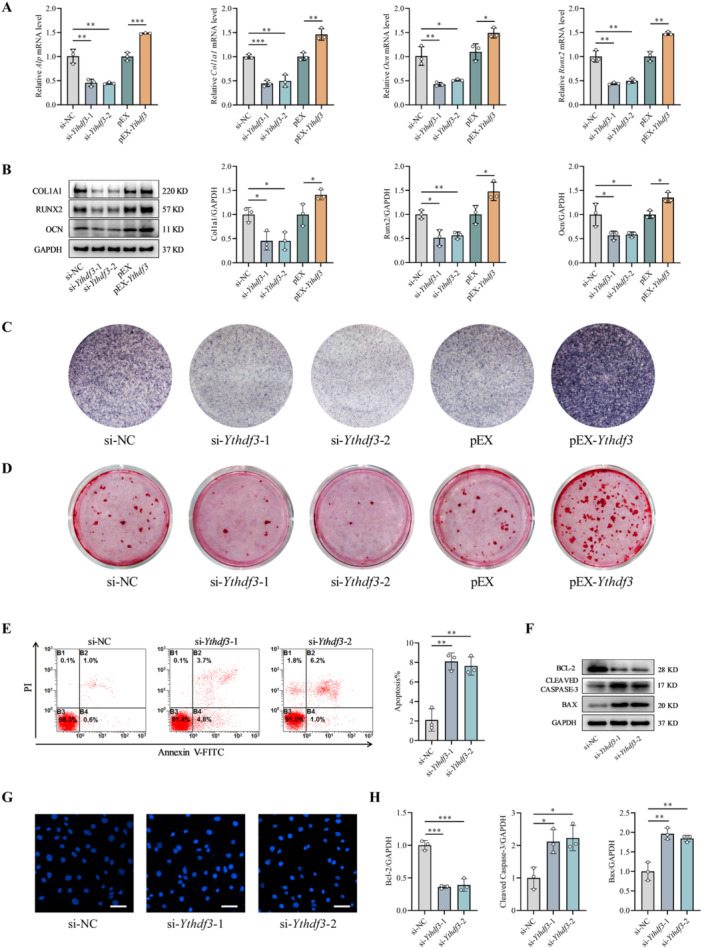
YTHDF3 orchestrates osteogenic progression by enhancing differentiation and mineralization while simultaneously inhibiting apoptosis. (A) Transcriptional regulation of osteogenic marker genes modulated by *Ythdf3* knockdown or overexpression (*n* = 3). (B) Protein abundance of key osteogenic differentiation regulators under YTHDF3 manipulation (*n* = 3). (C) Representative ALP staining image demonstrating osteogenic differentiation capacity (*n* = 3). (D) Characteristic ARS staining image revealing the extent of matrix mineralization (*n* = 3). (E) Quantitative assessment of apoptosis via flow cytometry after *Ythdf3* depletion (*n* = 3). (F and H) Western blot analysis of apoptosis‐related effectors, including proapoptotic proteins (Bax and cleaved caspase‐3) and antiapoptotic Bcl‐2 (*n* = 3). (G) Morphological assessment of apoptotic osteoblasts by Hoechst 33 258 nuclear staining (*n* = 3). Scale bar, 50 µm. *n* = 3 represents three independent biological replicates. *p < 0.05, **p < 0.01, ***p < 0.001 versus control.

To elucidate the functional significance of YTHDF3 in osteoblasts, we implemented optimized knockdown and overexpression approaches in MC3T3‐E1 cells. Transfection efficiency was quantified by flow cytometry (Figure S5C), with both qRT–PCR and Western blot analyses confirming robust *Ythdf3* knockdown using si‐*Ythdf3*‐1 and si‐*Ythdf3*‐2 and efficient overexpression via the pEX‐*Ythdf3* plasmid (Figure S5D,E). Functional characterization revealed that *Ythdf3* depletion substantially attenuated the expression of key osteogenic markers (ALP, COL1A1, OCN, and RUNX2) at both the transcriptional and protein levels, whereas *Ythdf3* overexpression strongly increased their expression (Figure 3A,B). In parallel, ALP and ARS staining revealed that *Ythdf3* deficiency compromised osteogenic differentiation and matrix mineralization capacity, whereas *Ythdf3* overexpression enhanced these processes (Figure 3C,D). Assessment of apoptosis through flow cytometry and Hoechst 33258 staining confirmed that *Ythdf3* knockdown increased apoptosis rates (Figure 3E,G), which was supported by Western blot data showing concomitant upregulation of proapoptotic effectors (Bax and cleaved caspase‐3) and downregulation of antiapoptotic Bcl‐2 (Figure 3F,H). These comprehensive analyses reveal that YTHDF3 coordinately regulates osteoblast differentiation and mineralization while exerting antiapoptotic effects, emphasizing its fundamental role in preserving osteoblast homeostasis.

### 3.5 Melatonin Alleviates Mechanical Unloading‐Induced Osteoblast Dysfunction via YTHDF3‐Dependent Mechanisms

**Figure 4:**
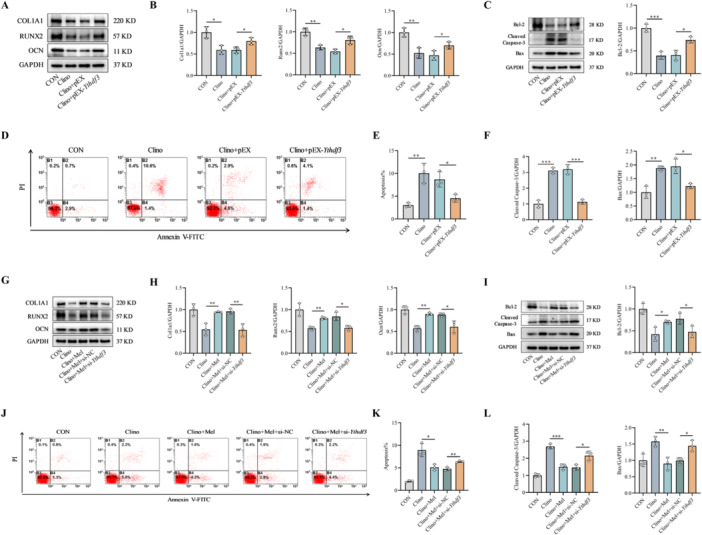
Melatonin alleviates mechanical unloading‐induced osteoblast dysfunction via a YTHDF3‐dependent mechanism. (A and B) Protein abundance of key osteogenic regulators in MC3T3‐E1 cells overexpressing *Ythdf3* and exposed to 48 h of clinorotation (*n* = 3). (C and F) Western blot evaluation of apoptosis regulators, including proapoptotic proteins (Bax and cleaved caspase‐3) and antiapoptotic Bcl‐2 (*n* = 3). (D and E) Quantitative assessment of apoptosis via flow cytometry in *Ythdf3*‐overexpressing MC3T3‐E1 cells after 48 h of mechanical unloading (*n* = 3). (G and H) Western blot analysis of key osteogenic differentiation proteins (*n* = 3). (I and L) Western blot evaluation of apoptosis‐related proteins, including proapoptotic effectors (Bax and cleaved caspase‐3) and antiapoptotic Bcl‐2 (*n* = 3). (J and K) Flow cytometric quantification of apoptosis (*n* = 3). *n* = 3 represents three independent biological replicates. *p < 0.05, **p < 0.01, ***p < 0.001 versus control.

To evaluate the potential of YTHDF3 in mitigating mechanical unloading‐induced osteoblast dysfunction, we transfected MC3T3‐E1 cells with pEX‐*Ythdf3* followed by 48 h of clinorotation. qRT–PCR analysis revealed that mechanical unloading (Clino group) significantly suppressed the mRNA expression of key osteogenic markers (*Alp*, *Col1a1*, *Ocn*, and *Runx2*) compared with that in the static control (CON) group (Figure S6A). Notably, *Ythdf3* overexpression (Clino + pEX‐*Ythdf3* group) substantially reversed this transcriptional repression compared with that in the negative control (Clino + pEX) group (Figure S6A). Western blot corroborated these findings at the protein level, revealing that the clinorotation‐induced reductions in COL1A1, RUNX2, and OCN expression were effectively reversed by *Ythdf3* overexpression (Figure 4A,B). Flow cytometric analysis further demonstrated that *Ythdf3* overexpression significantly ameliorated the increase in the apoptosis rate caused by mechanical unloading (Figure 4D,E). Consistent with these findings, Western blot revealed that both the unloading‐induced upregulation of proapoptotic proteins (Bax and cleaved caspase‐3) and the downregulation of antiapoptotic Bcl‐2 were counteracted by *Ythdf3* overexpression (Figure 4C,F). These collective results establish that YTHDF3 partially alleviates mechanical unloading‐induced osteoblast dysfunction through enhancing differentiation and suppressing apoptosis.

Given that melatonin rescued mechanical unloading‐induced YTHDF3 downregulation and that YTHDF3 intrinsically protected osteoblast function under unloading conditions, we proceeded to determine whether YTHDF3 mediates the protective effects of melatonin. MC3T3‐E1 cells were transfected with si‐*Ythdf3* and cultured under 48 h of clinorotation with melatonin supplementation. qRT–PCR analysis revealed that melatonin significantly counteracted the unloading‐induced suppression of *Alp*, *Col1a1*, *Ocn*, and *Runx2* transcription, an effect that was substantially abrogated by *Ythdf3* knockdown (Figure S6B). Western blot verified that melatonin‐mediated restoration of COL1A1, OCN, and RUNX2 protein expression under unloading conditions was similarly compromised upon *Ythdf3* depletion (Figure 4G,H). Flow cytometric analysis confirmed that melatonin ameliorated the increase in the apoptosis rate induced by mechanical unloading, whereas *Ythdf3* knockdown significantly attenuated this protective effect (Figure 4J,K). Similarly, Western blot analysis demonstrated that melatonin reversed the unloading‐induced upregulation of proapoptotic proteins (Bax and cleaved caspase‐3) and downregulation of antiapoptotic Bcl‐2, effects that were largely nullified by *Ythdf3* knockdown (Figure 4I,L). We therefore concluded that melatonin ameliorated mechanical unloading‐induced osteoblast dysfunction primarily through the upregulation of YTHDF3 expression.

### 3.6 Bone‐Directed *Ythdf3* Gene Delivery Demonstrates Partial Therapeutic Efficacy Against Disuse Osteoporosis in HLU Mice

**Figure 5:**
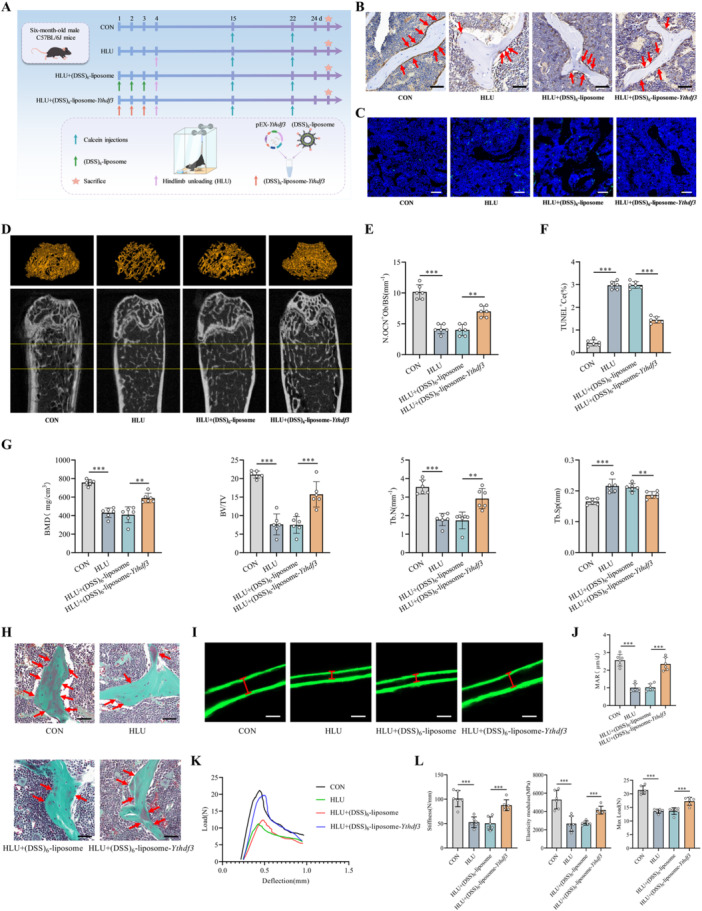
Bone‐directed *Ythdf3* delivery demonstrates partial therapeutic efficacy against disuse osteoporosis in HLU mice. (A) Schematic diagram of the experimental design. (B) Representative images of IHC staining for OCN in the distal femur. Scale bar, 50 μm. (C) Representative images of TUNEL staining of the distal femur. Scale bar, 50 μm. (D) Representative 3D micro‐CT reconstruction images of the ROI in the distal femur. (E) Quantitative analysis of OCN‐positive osteoblast numbers (*n* = 6). (F) Quantitative analysis of TUNEL‐positive cell percentages (*n* = 6). (G) Quantitative analysis of bone parameters, including the BMD, BV/TV, Tb.N, and Tb.Sp (*n* = 6). (H) Goldner's trichrome staining of the distal femur, distinguishing osteoid (red) from mineralized bone (green) for assessing new bone formation (*n* = 6). Scale bar, 50 μm. (I) Representative images of calcein double‐labeling for dynamic histomorphometric analysis of bone formation. Scale bar, 20 μm. (J) Quantitative analysis of the mineral apposition rate (MAR) (*n* = 6). (K) Representative load–displacement curves obtained from three‐point bending tests. (L) Quantitative analysis of biomechanical parameters, including stiffness, maximum load, and elastic modulus (*n* = 6). **p < 0.01, ***p < 0.001 versus control.

Our investigation revealed consistent downregulation of YTHDF3 expression under mechanical unloading conditions across both cellular and animal models. Through mechanistic studies, we determined that YTHDF3 overexpression effectively ameliorates unloading‐induced osteoblast dysfunction and demonstrated that the protective effects of melatonin against mechanical unloading are predominantly mediated through the upregulation of YTHDF3 expression. To further evaluate the therapeutic potential of YTHDF3 in preventing mechanical unloading‐induced bone loss, we employed a targeted delivery strategy utilizing a (DSS)_6_‐liposome system for the site‐specific administration of a *Ythdf3* overexpression plasmid to bone formation sites in mice. Systematic validation confirmed both efficacy and safety: Western blot and qRT–PCR revealed that bone‐targeted *Ythdf3* delivery significantly increased YTHDF3 expression in bone tissue (Figure S6C,D). Moreover, H&E staining revealed no significant pathological alterations or tissue damage in organs from (DSS)_6_‐liposome‐*Ythdf3*‐treated mice (Figure S6E), confirming the biosafety of this targeted approach. In subsequent therapeutic experiments, the mice were randomly allocated to four experimental groups: CON, HLU, HLU + (DSS)_6_‐liposome, and HLU + (DSS)_6_‐liposome‐*Ythdf3* (Figure 5A). After three consecutive days of (DSS)_6_‐liposome‐*Ythdf3* administration and 21 days of subsequent HLU, we observed significant downregulation of YTHDF3 expression at both the transcriptional and translational levels in HLU mice compared with the YTHDF3 levels in the CON group (Figure S6F,G). Crucially, bone‐targeted *Ythdf3* delivery successfully restored YTHDF3 expression in the HLU + (DSS)_6_‐liposome‐*Ythdf3* group (Figure S6F,G).

IHC evaluation of bone sections revealed a substantial reduction in osteoblast numbers along bone surfaces in HLU mice compared with normal controls (CON) (Figure 5B,E). Bone‐targeted *Ythdf3* delivery effectively restored osteoblast populations, significantly increasing their numbers relative to those in untreated HLU mice (Figure 5B,E). Complementing these findings, TUNEL staining revealed a pronounced increase in the percentage of apoptotic cells in the HLU group, which was substantially attenuated in mice receiving (DSS)_6_‐liposome‐*Ythdf3* treatment compared with those administered empty (DSS)_6_‐liposome vectors (Figure 5C,F). These data establish that targeted *Ythdf3* delivery effectively promotes osteoblast preservation and inhibits apoptosis under mechanical unloading conditions. Quantitative assessments of bone mass and microarchitectural parameters within defined regions of interest were performed via micro‐CT analysis of femoral specimens. Three‐dimensional reconstructions illustrated that compared with control conditions, HLU induced characteristic osteoporotic transformation of the trabecular architecture (Figure 5D). Notably, bone‐directed *Ythdf3* supplementation substantially prevented this unloading‐induced deterioration of trabecular structure (Figure 5D). Quantitative morphometric analysis confirmed that mechanical unloading significantly compromised bone quality parameters, reducing the BMD, BV/TV, and Tb.N while increasing Tb.Sp (Figure 5G). Crucially, bone‐targeted *Ythdf3* administration effectively reversed these pathological changes, significantly increasing the BMD, BV/TV, and Tb.N while reducing Tb.Sp (Figure 5G). Moreover, TRAP staining revealed abundant quantities of TRAP‐positive osteoclasts in HLU mice and markedly reduced amounts in YTHDF3‐treated mice (Figure S6H). These comprehensive analyses revealed that YTHDF3 represents an effective therapeutic target for counteracting mechanical unloading‐induced bone loss and preserving trabecular microarchitecture in disuse osteoporosis models.

Bone formation was comprehensively evaluated using Goldner's trichrome staining and calcein double‐labeling techniques. Goldner's staining demonstrated substantial impairment of new bone formation in HLU mice, which was effectively reversed by bone‐targeted *Ythdf3* supplementation (Figure 5H). Dynamic histomorphometric assessment via calcein double‐labeling revealed a significantly reduced MAR in HLU mice, indicating suppressed osteogenic activity (Figure 5I,J). Importantly, bone‐directed *Ythdf3* delivery substantially increased the MAR (Figure 5I,J), indicating strong stimulation of bone formation. Biomechanical properties were examined using three‐point bending tests, with representative load–displacement curves showing markedly compromised bone strength in HLU mice that was significantly improved following the *Ythdf3* intervention (Figure 5K). Quantitative analysis confirmed significant deterioration in key biomechanical parameters—including stiffness, maximum load, and elastic modulus—in HLU mice, all of which were effectively restored by bone‐targeted *Ythdf3* treatment (Figure 5L). These integrated findings establish that targeted *Ythdf3* delivery promotes bone formation and improves biomechanical competence under mechanical unloading conditions.

### 3.7 YTHDF3 Can Regulate DAPK2 Expression by Binding to a Specific m^6^A Motif in *Dapk2* mRNA

**Figure 6:**
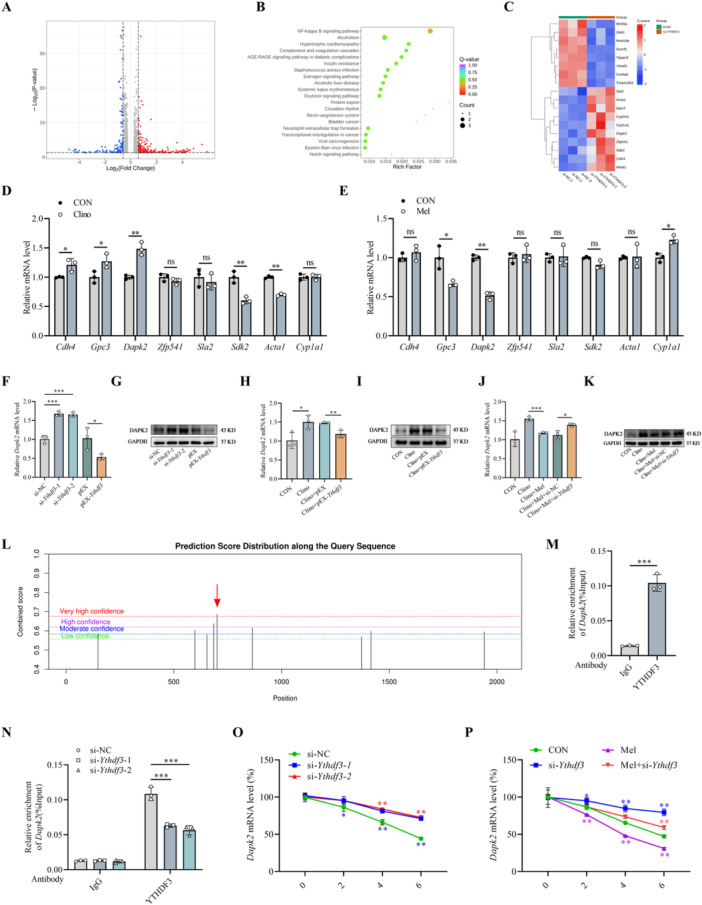
YTHDF3 could regulate DAPK2 expression by binding to a specific m^6^A motif in *Dapk2* mRNA. (A and B) Volcano plot and KEGG pathway analysis of differentially expressed mRNAs after transfection with si‐NC or si‐*Ythdf3*. (C) Heatmap based on RNA‐seq data. (D) Transcript levels of candidate targets quantified by qRT–PCR in MC3T3‐E1 cells subjected to mechanical unloading (*n* = 3). (E) Parallel qRT‐PCR assessment to determine the mRNA expression patterns of candidate factors in melatonin‐treated osteoblasts (*n* = 3). (F) Transcriptional regulation of *Dapk2* by YTHDF3 manipulation, as quantified by qRT–PCR, in MC3T3‐E1 cells transfected with either si‐*Ythdf3* or pEX‐*Ythdf3* (*n* = 3). (G) Corresponding modulation of DAPK2 protein expression assessed by Western blot analysis following *Ythdf3* knockdown or overexpression (*n* = 3). (H) qRT–PCR‐based quantification of *Dapk2* mRNA expression in *Ythdf3*‐overexpressing MC3T3‐E1 cells under mechanical unloading (*n* = 3). (I) Western blot analysis of DAPK2 protein levels in *Ythdf3*‐overexpressing MC3T3‐E1 cells exposed to clinorotation (*n* = 3). (J) *Dapk2* transcript levels measured by qRT–PCR in *Ythdf3*‐deficient MC3T3‐E1 cells treated with melatonin during 48 h of mechanical unloading (*n* = 3). (K) Western blot analysis of DAPK2 protein expression in *Ythdf3*‐silenced MC3T3‐E1 cells maintained in melatonin‐supplemented medium under clinorotation (*n* = 3). (L) The online SRAMP database revealed the multiple possible m^6^A modification sites within the *Dapk2* transcript. (M and N) RIP‐PCR analysis confirming specific binding between YTHDF3 protein and *Dapk2* mRNA (*n* = 3). (O) RNA stability evaluation revealing that YTHDF3 deficiency significantly decreased *Dapk2* mRNA decay rates (*n* = 3). (P) RNA stability studies confirming that melatonin potentiated *Dapk2* mRNA degradation in a YTHDF3‐dependent manner (*n* = 3). *n* = 3 represents three independent biological replicates. *p < 0.05, **p < 0.01, ***p < 0.001 versus control. ns = not significant.

To elucidate the molecular mechanisms underlying the YTHDF3‐mediated regulation of osteoblast differentiation, mineralization, and apoptosis, we performed transcriptomic profiling through RNA‐seq using MC3T3‐E1 cells transfected with either si‐NC or si‐*Ythdf3*. This comprehensive analysis aimed to identify critical target genes and signaling pathways through which YTHDF3 orchestrates osteoblast function. Comparative transcriptome analysis revealed 238 significantly upregulated genes and 117 significantly downregulated genes. Genome‐wide expression patterns and pathway enrichments were visualized through volcano plot representation and KEGG pathway analysis (Figure 6A,B), while heatmaps specifically highlighted differentially expressed genes associated with bone metabolism‐related pathways (Figure 6C).

Due to the well‐characterized function of YTHDF3 as an m^6^A reader protein that recognizes m^6^A modifications and facilitates mRNA decay, we initially focused on upregulated candidate transcripts and subsequently refined our selection to genes exhibiting coordinated regulation by both melatonin and mechanical unloading. Considering the antagonistic regulation of YTHDF3 expression by mechanical unloading (suppressive) versus melatonin (inductive), we specifically prioritized genes whose expression was upregulated under unloading conditions but downregulated in response to melatonin. Through qRT–PCR validation, *Dapk2* emerged as the most strikingly upregulated transcript under mechanical unloading and the most potently suppressed transcript under melatonin treatment (Figure 6D,E), thus warranting its selection for detailed mechanistic exploration. A functional assessment revealed that *Ythdf3* overexpression substantially suppressed both DAPK2 protein and mRNA expression, whereas *Ythdf3* knockdown conversely increased DAPK2 levels (Figures 6F,G and S7A). Importantly, *Ythdf3* overexpression in MC3T3‐E1 cells effectively neutralized the mechanical unloading‐induced upregulation of DAPK2 expression (Figures 6H,I and S7B). Corroborating these cellular observations, HLU mice displayed elevated DAPK2 expression in the hindlimb tibia, which was effectively normalized by (DSS)_6_‐liposome‐mediated *Ythdf3* delivery (Figure S7D,E). To elucidate the potential involvement of YTHDF3 in melatonin‐mediated DAPK2 regulation under unloading conditions, we conducted *Ythdf3* knockdown experiments combined with melatonin treatment during clinorotation. Melatonin administration significantly ameliorated the unloading‐induced increase in DAPK2 elevation, an effect that was substantially compromised upon *Ythdf3* depletion (Figures 6J,K and S7C), demonstrating that the regulatory effect of melatonin on DAPK2 expression is partially mediated through YTHDF3.

To elucidate the molecular mechanism underlying the YTHDF3‐mediated regulation of *Dapk2* expression, we investigated whether YTHDF3 directly associates with m^6^A modification sites on the *Dapk2* transcript and modulates its stability. Subcellular localization studies confirmed the predominant cytoplasmic localization of YTHDF3 in MC3T3‐E1 cells (Figure S7F,G), consistent with its established function in posttranscriptional regulation. RNA stability assessment demonstrated that *Ythdf3* knockdown significantly prolonged the half‐life of *Dapk2* mRNA (Figure 6O). Furthermore, melatonin treatment accelerated *Dapk2* mRNA decay, an effect that was substantially attenuated in *Ythdf3*‐deficient cells, indicating that melatonin potentiates mRNA degradation through a YTHDF3‐dependent mechanism (Figure 6P). Bioinformatic analysis using the SRAMP algorithm predicted multiple conserved m^6^A modification sites within *Dapk2* mRNA (Figure 6L). RIP‐PCR analysis confirmed the direct binding of YTHDF3 to m^6^A‐modified regions of the *Dapk2* transcript, with significantly enriched binding signals that were markedly reduced upon *Ythdf3* knockdown (Figures 6M,N and S7H). Importantly, global m^6^A methylation levels remained unaltered following *Ythdf3* depletion (Figure S7I), and MeRIP‐qPCR revealed no significant changes in the abundance of m^6^A modifications on *Dapk2* mRNA upon *Ythdf3* knockdown (Figure S7J), demonstrating that YTHDF3 functions as a recognition protein rather than a modulator of m^6^A methylation. Subsequently, we replaced the *N* ^6^‐methylated adenosine (A) sites in the CDS region of DAPK2 that were effective in the RIP assay with thymine (T), generating the DAPK2 mutant (MUT) (Figure S7K). The subsequent dual‐luciferase reporter gene assay demonstrated that YTHDF3 significantly inhibited the luciferase activity of the wild‐type construct but had no effect on MUT (Figure S7L). These comprehensive findings establish that YTHDF3 regulates *Dapk2* mRNA degradation in an m^6^A‐dependent manner, thereby revealing a crucial posttranscriptional regulatory axis involved in the maintenance of osteoblast homeostasis.

### 3.8 Melatonin Enhances Osteoblast Differentiation and Suppresses Apoptosis Under Mechanical Unloading via the YTHDF3/DAPK2 Pathway

**Figure 7:**
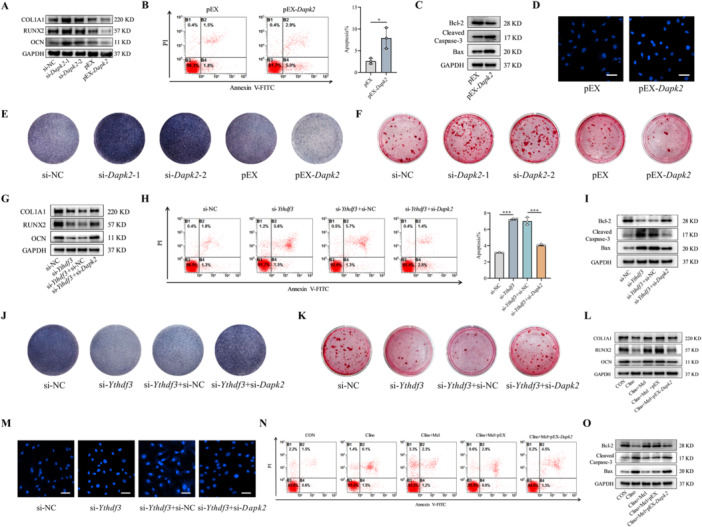
Melatonin enhances osteoblast differentiation and suppresses apoptosis under mechanical unloading via the YTHDF3/DAPK2 pathway. (A) Western blot analysis of key osteogenic differentiation proteins modulated by *Dapk2* manipulation (*n* = 3). (B) Quantitative assessment of apoptosis after *Dapk2* overexpression via flow cytometry (*n* = 3). (C) Western blot analysis of apoptosis‐related effectors, including proapoptotic proteins (Bax and cleaved caspase‐3) and antiapoptotic Bcl‐2 (*n* = 3). (D) Morphological assessment of apoptotic osteoblasts by Hoechst 33258 nuclear staining (*n* = 3). Scale bar, 50 µm. (E) Representative ALP staining image demonstrating osteogenic differentiation capacity (*n* = 3). (F) Representative ARS staining image indicating osteoblast mineralization capacity (*n* = 3). (G) Western blot analysis of key osteogenic differentiation regulators following dual transfection with si‐*Ythdf3* and si‐*Dapk2* in MC3T3‐E1 cells (*n* = 3). (H) Flow cytometric quantification of apoptosis following dual transfection with si‐*Ythdf3* and si‐*Dapk2* in MC3T3‐E1 cells (*n* = 3). (I) Western blot evaluation of apoptosis‐related proteins Bax, cleaved caspase‐3, and Bcl‐2 following dual transfection with si‐*Ythdf3* and si‐*Dapk2* in MC3T3‐E1 cells (*n* = 3). (J) Representative ALP staining image demonstrating osteogenic capacity following dual transfection with si‐*Ythdf3* and si‐*Dapk2* in MC3T3‐E1 cells (*n* = 3). (K) Representative ARS staining image revealing mineralization potential following dual transfection with si‐*Ythdf3* and si‐*Dapk2* in MC3T3‐E1 cells (*n* = 3). (L) Following transfection with pEX‐*Dapk2*, MC3T3‐E1 cells were maintained in melatonin‐supplemented medium for 48 h of clinorotation. Western blot evaluation of key osteogenic regulatory proteins (*n* = 3). (M) Morphological assessment of apoptotic nuclei by Hoechst 33258 staining following dual transfection with si‐*Ythdf3* and si‐*Dapk2* in MC3T3‐E1 cells (*n* = 3). Scale bar, 50 µm. (N) Following transfection with pEX‐*Dapk2*, MC3T3‐E1 cells were maintained in melatonin‐supplemented medium for 48 h of clinorotation. Flow cytometric quantification of apoptosis (*n* = 3). (O) Following transfection with pEX‐*Dapk2*, MC3T3‐E1 cells were maintained in melatonin‐supplemented medium for 48 h of clinorotation. Western blot assessment of the apoptosis‐related proteins Bax, cleaved caspase‐3, and Bcl‐2 (*n* = 3). *n* = 3 represents three independent biological replicates. *p < 0.05, ***p < 0.001 versus control.

To functionally characterize the role of DAPK2 as a downstream effector of YTHDF3 in regulating osteoblast activity, we precisely modulated DAPK2 expression in MC3T3‐E1 cells through siRNA‐mediated knockdown and plasmid‐based overexpression approaches (Figure S7M,N). Genetic silencing of *Dapk2* expression significantly increased the transcription levels of the essential osteogenic markers *Alp*, *Col1a1*, *Ocn*, and *Runx2* (Figure S8A), with corresponding increases in the expression of their protein products COL1A1, OCN, and RUNX2 (Figure 7A; S8B). Conversely, *Dapk2* overexpression substantially suppressed both the mRNA and protein expression of these osteogenic factors (Figures 7A and S8A,B). Functional assessments through ALP and ARS staining consistently demonstrated that *Dapk2* depletion increased osteogenic differentiation and matrix mineralization, whereas *Dapk2* overexpression impaired these processes (Figure 7E,F). Apoptosis analysis revealed that *Dapk2* overexpression significantly increased osteoblast apoptosis rates, as quantified by flow cytometry (Figure 7B), with complementary Hoechst 33258 staining confirming this proapoptotic effect (Figure 7D). Mechanistically, *Dapk2* overexpression upregulated the key proapoptotic mediators Bax and cleaved caspase‐3 while downregulating the antiapoptotic protein Bcl‐2 (Figures 7C and S8C). These comprehensive data establish DAPK2 as a negative regulator of osteoblast differentiation and mineralization and a positive regulator of osteoblast apoptosis.

To elucidate the functional relationship between YTHDF3 and DAPK2 in osteoblast regulation, we conducted combinatorial gene knockdown studies in MC3T3‐E1 cells using si‐*Ythdf3* and si‐*Dapk2*. Individual *Ythdf3* knockdown significantly suppressed the mRNA expression of key osteogenic markers (*Alp*, *Col1a1*, *Ocn*, and *Runx2*) and reduced the corresponding protein levels (COL1A1, RUNX2, and OCN) (Figures S8D,E and 7G). Notably, concomitant *Dapk2* knockdown substantially reversed this *Ythdf3* deficiency‐mediated suppression, effectively restoring both the transcriptional and translational expression of these differentiation markers (Figures S8D,E and 7G). Functional evaluation through ALP and ARS staining further confirmed that *Dapk2* depletion rescued the compromised osteogenic differentiation and mineralization capacity resulting from *Ythdf3* knockdown (Figure 7J,K). Flow cytometric analysis revealed that *Ythdf3* deficiency increased osteoblast apoptosis rates, whereas concurrent *Dapk2* knockdown effectively attenuated this apoptotic response (Figure 7H). This antiapoptotic effect was corroborated by Hoechst 33258 staining, which revealed consistent morphological changes (Figure 7M). Mechanistic investigations revealed that *Ythdf3* knockdown promoted apoptosis through the upregulation of the expression of the proapoptotic proteins Bax and cleaved caspase‐3 and the downregulation of the expression of the antiapoptotic protein Bcl‐2 (Figures 7I and S8F). Critically, combined *Dapk2* knockdown counteracted these proapoptotic alterations, significantly reducing apoptosis rates while normalizing the expression balance between pro‐ and antiapoptotic regulators (Figures 7I and S8F). These comprehensive findings establish DAPK2 as an essential downstream mediator of the regulatory effects of YTHDF3 on osteoblast differentiation, mineralization, and survival.

Previous investigations demonstrated that melatonin upregulates YTHDF3 expression to promote osteoblast differentiation and inhibit apoptosis under mechanical unloading, whereas YTHDF3 modulates osteoblast differentiation, mineralization, and apoptosis through DAPK2. To further elucidate the functional relationship between melatonin and DAPK2 in this context, we transfected MC3T3‐E1 cells with pEX‐*Dapk2* and treated them with melatonin during 48 h of clinorotation. Melatonin mitigated the suppressive effects of mechanical unloading on osteoblast differentiation (Figures S8G,I and 7L). However, when *Dapk2* was overexpressed, melatonin failed to reverse the impairment of osteogenic differentiation induced by unloading (Figures S8G,I and 7L). Flow cytometric and Western blot analyses further revealed that *Dapk2* overexpression abolished the ability of melatonin to attenuate unloading‐induced apoptosis (Figures 7N,O and S8H,J). These findings indicate that melatonin regulates osteoblast function through DAPK2 under mechanical unloading. Mechanistically, melatonin enhances YTHDF3 expression, which in turn downregulates DAPK2 expression. Collectively, these results demonstrate that melatonin promotes osteoblast differentiation and inhibits apoptosis under mechanical unloading through the YTHDF3/DAPK2 signaling axis.

## 4 Discussion

Mechanical unloading‐associated bone loss represents a prevalent clinical condition affecting individuals with extended activity restriction, particularly long‐term bedridden patients and astronauts, that has stimulated considerable scientific investigation. Substantial evidence indicates that mechanical unloading disrupts skeletal homeostasis by impairing bone formation while simultaneously enhancing bone resorption, collectively driving progressive bone mass depletion. Osteogenesis is principally orchestrated by osteoblasts [3, 4, 32], which function as mechanosensory cells capable of detecting mechanical cues and transducing them into biochemical signals that modulate gene expression programs [33]. Despite these advances, the complete molecular circuitry governing mechanical unloading‐induced osteopenia remains to be fully elucidated. Contemporary therapeutic approaches for counteracting unloading‐mediated bone loss involve mainly exercise regimens and pharmacological treatments. Although these interventions provide modest benefits in slowing bone loss progression, they have several limitations, including suboptimal efficacy, significant adverse effects associated with drug therapies, and poor long‐term medication compliance among patients.

Melatonin serves as a crucial regulator in preserving bone and cartilage homeostasis. Accumulating evidence has shown that melatonin significantly mitigates bone loss in clinical postmenopausal populations and experimental models, including estrogen‐deficient and aged mice [34, 35]. Recent studies have demonstrated that melatonin attenuates clinostat‐induced autophagic flux in MC3T3‐E1 cells [36], whereas under mechanical unloading conditions, it curbs osteoclast‐mediated bone resorption by modulating key regulators through calcitonin upregulation and RANKL downregulation [37]. Capitalizing on its advantageous profile featuring low production costs and limited side effects, melatonin represents a viable prophylactic and therapeutic option for disuse osteoporosis. In our experimental approach, we initially implemented a 2D clinostat system to establish a robust osteoblast mechanical unloading model, confirming that melatonin effectively enhanced osteogenic differentiation capacity while concurrently inhibiting apoptosis under unloading‐induced stress. Through complementary HLU murine experiments, we demonstrated that exogenous melatonin supplementation substantially increased osteoblast numbers in bone tissue, decreased apoptosis rates, improved BMD and trabecular microarchitecture, stimulated new bone formation, and enhanced bone mechanical properties. The intricate molecular mechanisms through which melatonin orchestrates osteoblast functional regulation and bone anabolic processes under mechanical unloading conditions, however, remain to be fully elucidated and represent important directions for future research.

Accumulating research has revealed that melatonin confers protection against pathological processes including vascular calcification, as well as in reproductive physiology and gestational maintenance, through the regulation of m^6^A RNA modifications, indicating that m^6^A‐mediated epitranscriptomic mechanisms significantly contribute to its therapeutic effects [18, 38, 39]. However, the interplay between melatonin signaling and m^6^A modifications in skeletal metabolism, especially under mechanical unloading conditions, represents an important knowledge gap.

Sophisticated epigenetic mechanisms—spanning DNA methylation patterns, histone posttranslational modifications, noncoding RNA networks, and RNA chemical modifications—have been identified as fundamental regulators of bone formation dynamics [40, 41]. Current epigenetic research focusing on mechanical unloading‐induced bone metabolic alterations has primarily emphasized noncoding RNAs and histone modifications (exemplified by the histone methyltransferase SETDB1, the histone deacetylase HDAC1, and various lncRNA/miRNA species) [42, 43, 44], while the contribution of RNA modifications to osteoblast functional adaptation and bone formation capacity under unloading conditions remains inadequately characterized. As the most abundant internal mRNA modification in eukaryotes, m^6^A adenosine methylation is involved extensively in RNA metabolic pathways, including those related to precursor mRNA processing, nuclear export, translational efficiency modulation, and RNA decay regulation, serving critical functions in diverse physiological and pathological contexts ranging from immunological disorders, neural development, and reproductive conditions to osteoporosis and malignant progression [45, 46, 47]. The regulatory significance of m^6^A modifications in bone metabolism and skeletal homeostasis has recently attracted substantial scientific interest. Cutting‐edge studies have demonstrated that the genetic ablation of METTL3 compromises osteogenic differentiation programs, causing defective bone formation and osteoporotic phenotypes [48]; METTL14 expression deficiency in osteoporotic human specimens and ovariectomized murine models suppresses osteogenic activity via diminished m^6^A methylation of *Tcf1* mRNA [49]; and FTO demethylase activity is indispensable for normal bone development and mineralization in murine systems, with its loss resulting in growth impairment and reduced bone mass [50]. While m^6^A writer and eraser proteins have received considerable attention, the reader proteins that interpret m^6^A methylation marks constitute equally vital components in decoding m^6^A functional outputs and understanding the pathomechanisms of osteoporosis.

YTH domain family proteins (YTHDF1, YTHDF2, and YTHDF3) constitute essential cytoplasmic RNA‐binding proteins that function as central interpreters of m^6^A modifications. YTHDF1 increases the translation efficiency of target mRNAs by engaging with translation initiation factors and facilitating ribosome loading [51]. YTHDF2 directs m^6^A‐modified transcripts to cytoplasmic processing bodies to accelerate their decay, resulting in stress‐responsive cellular redistribution patterns [52]. YTHDF3 demonstrates functional coordination with both YTHDF1 and YTHDF2 through shared mRNA targets and displays dual regulatory effects in terms of both translational enhancement and degradation promotion [24]. Contrasting evidence also suggests context‐dependent roles for YTHDF3 in RNA stabilization processes. The comprehensive mechanistic framework and functional specificity of YTHDF3 within m^6^A‐mediated regulatory networks remain to be fully delineated. While the role of YTHDF3 has been primarily investigated in oncological contexts and fibrotic disorders involving hepatic and cardiac systems [25], its role in skeletal biology has been minimally explored. The pioneering work by Feng et al. revealed that YTHDF3 enhances osteoblast proliferation and differentiation programs through *Sox4* mRNA stabilization [29]. However, the contributions of YTHDF3 to adaptive osteoblast responses under mechanical unloading conditions, along with its potential functional relationships with melatonin‐mediated signaling pathways, represent significant knowledge gaps that require systematic investigation.

Our research implemented complementary 2D clinorotation and HLU model systems to mimic mechanical unloading conditions. We discovered that the m^6^A reader protein YTHDF3 was significantly downregulated in both mechanically stimulated osteoblasts and skeletal tissues from HLU mice, whereas melatonin administration effectively reversed this suppression. Functional characterization revealed that YTHDF3 enhanced osteogenic differentiation and matrix mineralization while inhibiting programmed cell death in osteoblasts. Genetic augmentation of YTHDF3 expression partially reversed unloading‐induced deficits in osteoblast function, and the targeted delivery of *Ythdf3* via (DSS)_6_‐liposomes to bone formation sites in HLU animals alleviated unloading‐mediated bone loss in vivo. These observations establish YTHDF3 as a positive regulator of osteogenesis and a potential therapeutic target for counteracting mechanical unloading‐induced bone deterioration. Importantly, *Ythdf3* depletion under unloading conditions markedly attenuated the pro‐osteogenic efficacy of melatonin, indicating that YTHDF3 is a crucial molecular determinant through which melatonin exerts its bone‐protective effects under mechanical challenge. Our findings illuminate a novel therapeutic paradigm wherein the melatonin‐mediated targeting of YTHDF3 may yield innovative treatment modalities for disuse‐related osteopenia. Key mechanistic insights emerging from our study revealed that the YTHDF3‐dependent increase in bone formation involved precise regulation of DAPK2 expression. Previous investigations have shown that YTHDF3 recognizes m^6^A modifications to accelerate transcript degradation. Our comprehensive transcriptomic profiling combined with RNA immunoprecipitation assays demonstrated that YTHDF3 directly engaged with m^6^A modification sites on *Dapk2* mRNA to promote its decay, leading to the subsequent downregulation of DAPK2 protein expression. Functional validation experiments confirmed that DAPK2 is a bona fide downstream effector of YTHDF3 that contributes to the coordinated regulation of osteogenic differentiation, matrix mineralization, and apoptosis in osteoblasts. However, the downstream signaling pathways mediating these effects remain insufficiently defined.

DAPK2 is a calcium/calmodulin‐regulated serine/threonine kinase belonging to the death‐associated protein kinase (DAPK) family, members of which share conserved catalytic domains and function as well‐established mediators of apoptosis [53]. As a negative regulator of mechanistic target of rapamycin complex 1 (mTORC1), DAPK2 directly binds and phosphorylates Raptor at Ser721, suppressing mTORC1 activity independently of AMPK and robustly inducing autophagy under stress conditions such as Ca^2+^ overload or amino acid starvation [54]. Physiological autophagy is cytoprotective, whereas excessive or sustained autophagy depletes survival factors, disrupts homeostasis, and triggers or sensitizes apoptosis. Thus, we speculate that DAPK2 promotes osteoblast apoptosis by suppressing mTORC1‐mediated excessive autophagy via autophagy–apoptosis crosstalk, which, together with noncanonical apoptotic pathways [55], ultimately compromises osteoblast survival. Moreover. mTORC1 signaling is central to skeletal development and osteogenic differentiation. Osteoprogenitor‐specific deletion of mTOR or Raptor recapitulates the cleidocranial dysplasia phenotype of *Runx2* haploinsufficiency [56]. Mechanistically, the mTOR–Raptor–S6K1 axis phosphorylates ERα, enabling its synergy with DLX5 to activate the *Runx2* enhancer and drive osteogenic differentiation [56]. Pro‐osteogenic factors (e.g., peptide drugs [57], sweroside [58]) upregulate *Runx2*, *Osx*, and *Ocn* via the mTORC1/p‑S6 pathway, whereas a low‑phosphate diet suppresses mTORC1, impairs osteogenesis, and promotes adipogenesis [59]. Based on these findings, we propose that DAPK2 inhibits mTORC1–Raptor signaling, thereby blocking transcriptional activation of *Runx2*, downregulating osteogenic genes, and suppressing osteoblast differentiation, thus exerting dual negative regulation on osteoblasts. Future studies will focus on delineating the role of mTORC1 in osteoblast differentiation and apoptosis under mechanical unloading, further establishing mTORC1 as a key downstream effector of DAPK2.

Despite these advances, several compelling scientific questions warrant further investigation. First, the molecular determinants responsible for mechanical unloading‐induced YTHDF3 suppression in osteoblasts remain incompletely characterized, calling for further dissection of the primary mechanosensory apparatus that initiates transcriptional responses to mechanical stimuli. Second, the precise signaling circuitry through which melatonin enhances YTHDF3 expression requires comprehensive mapping to elucidate the upstream regulatory mechanisms involved. Finally, given that melatonin traditionally modulates osteoblast physiology through established bone metabolic pathways, determining potential functional integration and synergistic crosstalk between the newly identified melatonin/YTHDF3/DAPK2 regulatory axis and conventional bone signaling networks represents an important direction for future research that could unveil novel therapeutic nodes for treating disuse osteoporosis.

**Figure 8:**
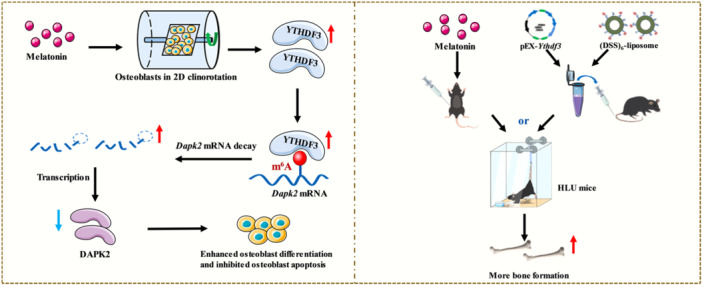
A schematic diagram illustrating the mechanism of melatonin/YTHDF3 in mitigating mechanical unloading‐induced bone loss. Melatonin regulates YTHDF3‐mediated m^6^A modification of Dapk2 mRNA, thereby protecting against bone loss caused by mechanical unloading.

In conclusion, our findings establish that melatonin counteracts mechanical unloading‐induced osteopenia by enhancing osteoblast differentiation capacity and inhibiting programmed cell death, ultimately preserving bone mass. We discovered that the m^6^A reader protein YTHDF3 is significantly downregulated under disuse conditions and that this suppression is effectively reversed by melatonin treatment. Genetic restoration of YTHDF3 expression partially reversed the unloading‐induced decrease in osteogenesis. Mechanistic interrogation revealed that YTHDF3 recognizes m^6^A modifications on *Dapk2* mRNA through direct binding, accelerating transcript decay and subsequent DAPK2 downregulation. Most significantly, we elucidated a previously unrecognized regulatory pathway in which melatonin orchestrates osteoblast differentiation and survival and bone formation under mechanical unloading through strategic modulation of the YTHDF3/DAPK2 signaling cascade, revealing new therapeutic targets for disuse osteoporosis (Figure 8).

## Author Contributions

All authors contributed to the study conception and design. Quan Sun, Liqun Xu, and Zhikui Li were responsible for material preparation, data collection, and analysis; Junfei Zhang, Xiran Zhao, Lijun Zhang, Xiaoyan Zhang, Jiangdong Zhao, Yingjun Tan, Luyao Wang, and Ge Zhang conducted investigation and software development; Zebing Hu, Shu Zhang, and Fei Shi oversaw supervision and funding acquisition; while Shu Zhang and Fei Shi led conceptualization, writing review, and editing. The initial manuscript draft was prepared by Quan Sun, with all authors providing critical feedback on subsequent revisions. All authors reviewed and endorsed the final manuscript.

## Ethics Statement

The experimental protocols involving animals were reviewed and approved by the Institutional Animal Care and Use Committee of Air Force Medical University (Approval No. IACUC‐20241247).

## Consent

All authors have reviewed the final manuscript, approved its submission, and consented to its publication.

## Conflicts of Interest

The authors declare no conflicts of interest.

## Supporting information

Supporting File

## Data Availability

The authors confirm that all data supporting the findings of this study are included within the article.
