# Supplementary material for: Melatonin Counteracts Mechanical Unloading‐Induced Bone Loss Through YTHDF3‐Mediated m6A Modification of Dapk2 mRNA
Source: J Pineal Res. 2026 May 18;78:e70146. doi: 10.1111/jpi.70146 (PMC13182765; doi:10.1111/jpi.70146)
Supplement: Supplementary file 1 — Supporting File [file JPI-78-e70146-s001.docx]

**Supplementary Materials for**

**Melatonin counteracts mechanical unloading-induced bone loss through YTHDF3-mediated m^6^A modification of *Dapk2* mRNA**

Quan Sun^1†^, Liqun Xu^1,2†^, Zhikui Li^1†^, Junfei Zhang^1^, Xiran Zhao^1^, Lijun Zhang^1,3^, Xiaoyan Zhang^1,2^, Jiangdong Zhao^1^, Yingjun Tan^5^, Luyao Wang^6^, Ge Zhang^6^, Zebing Hu^1*^, Shu Zhang^1*^ and Fei Shi^1*^

^*^To whom correspondence should be addressed: zebinghu@fmmu.edu.cn (Zebing Hu); shuzhang@fmmu.edu.cn (Shu Zhang); shifei719@fmmu.edu.cn (Fei Shi).

^1^The Key Laboratory of Aerospace Medicine, Ministry of Education, The Fourth Military Medical University, 710032, Xi’an, Shanxi, China

^2^Department of Gastroenterology and Endocrinology, Western Theater Air Force Hospital of PLA, 610065, Chengdu, Sichuan, China

^3^Department of Otolaryngology Head and Neck Surgery, Bethune International Peace Hospital, 050081, Shijiazhuang, Hebei, China

^4^Department of Otolaryngology Head and Neck Surgery, Western Theater Air Force Hospital of PLA, 610065, Chengdu Sichuan, China

^5^State Key Laboratory of Space Medicine Fundamentals and Application, China Astronaut Research and Training Center, 100094, Beijing, China

^6^Institute for Advancing Translational Medicine in Bone & Joint Diseases, School of Chinese Medicine, Hong Kong Baptist University, Hong Kong SAR, China

**Figure S1**

**
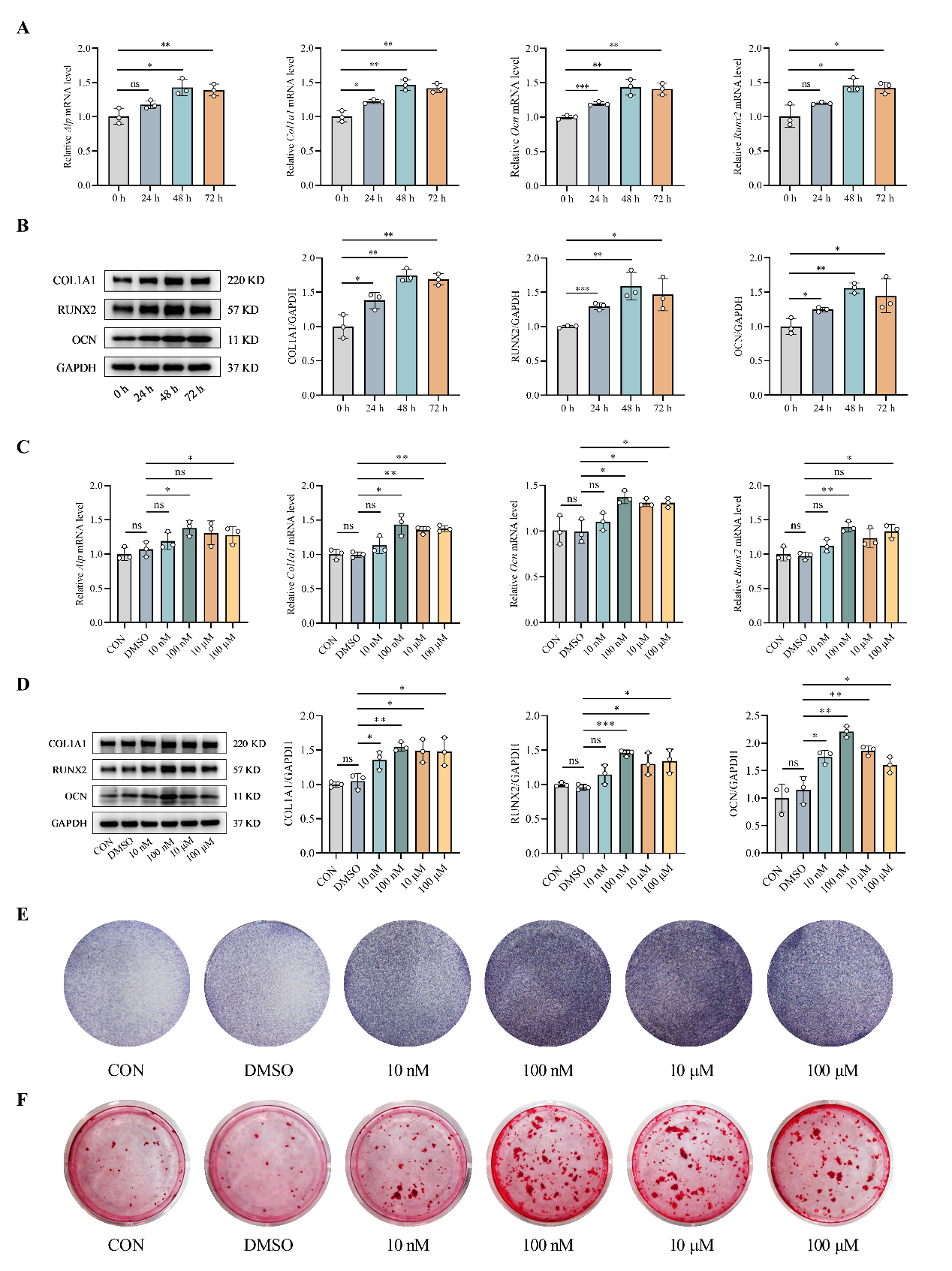
**

**Figure S1.** Screening of melatonin concentration and treatment duration to identify optimal conditions for osteogenic differentiation. **(A)** mRNA expression levels of the osteogenic markers *Alp*, *Col1a1*, *Ocn*, and *Runx2* in MC3T3-E1 cells treated with melatonin for 24, 48, and 72 hours (n=3). **(B)** Protein expression levels of COL1A1, RUNX2, and OCN under the same treatment conditions as in (A) (n=3). **(C)** mRNA expression of *Alp*, *Col1a1*, *Ocn*, and *Runx2* in cells treated with a gradient of melatonin concentrations (10 nM, 100 nM, 1 μM, and 100 μM) for 48 hours (n=3). **(D)** Protein expression levels of COL1A1, RUNX2, and OCN corresponding to the treatments in (C) (n=3). **(E)** Representative images of ALP staining (n=3). **(F)** Representative images of ARS staining (n=3). n=3 represents three independent biological replicates. ^*^*P* < 0.05, ^**^*P* < 0.01, ^***^*P* < 0.001 vs. control; ns=not significant.

**Figure S2**


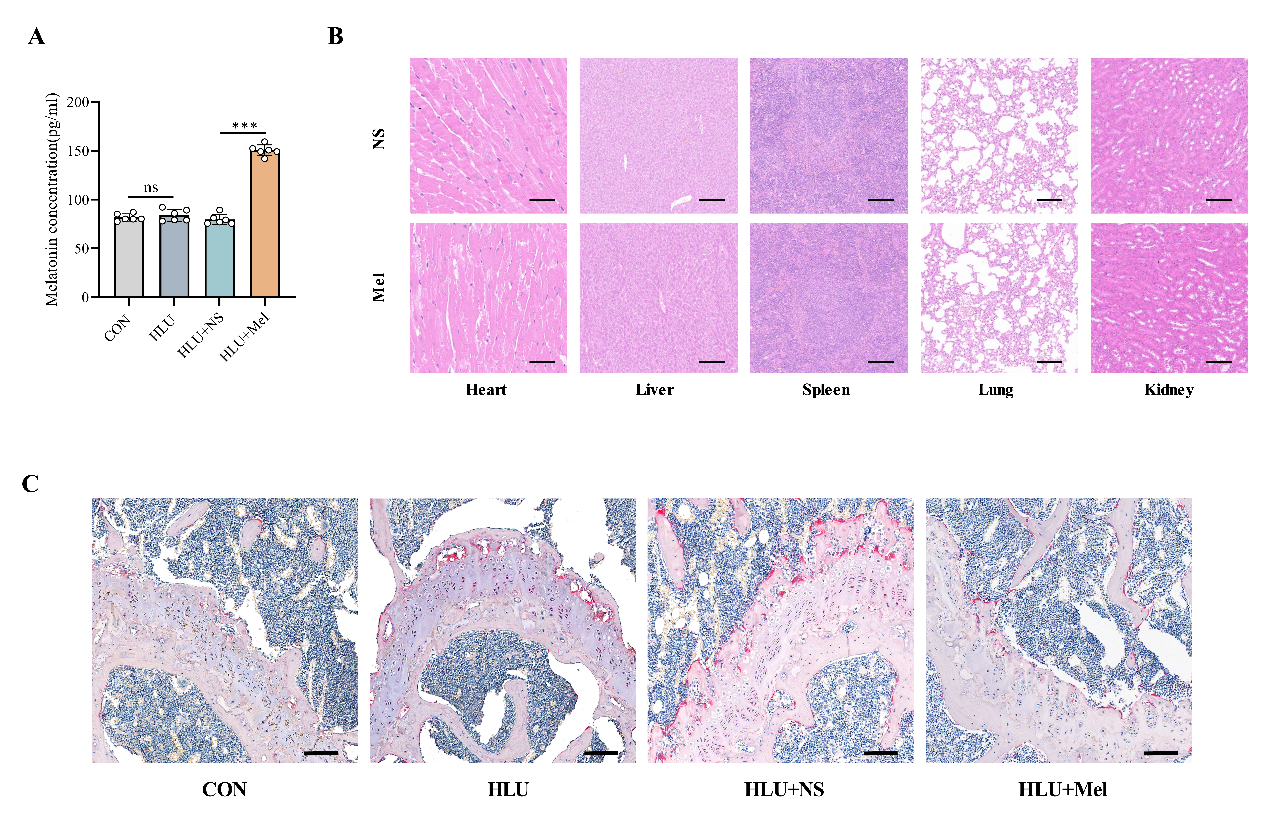


**Figure S2. (A)** Circulating melatonin concentrations in murine serum samples (n=6). **(B)** Histopathological assessment by H&E staining demonstrating the tissue architecture of major organs (heart, liver, spleen, lung, and kidney) following treatment with either normal saline (NS) or melatonin (Mel) (n=6). Scale bars, 100 μm. **(C)** TRAP staining was used to examine osteoclast numbers and activity in murine tissue with femur bone. Scale bars, 100 μm. ^***^*P* < 0.001 vs. control; ns=not significant.

**Figure S3**

**
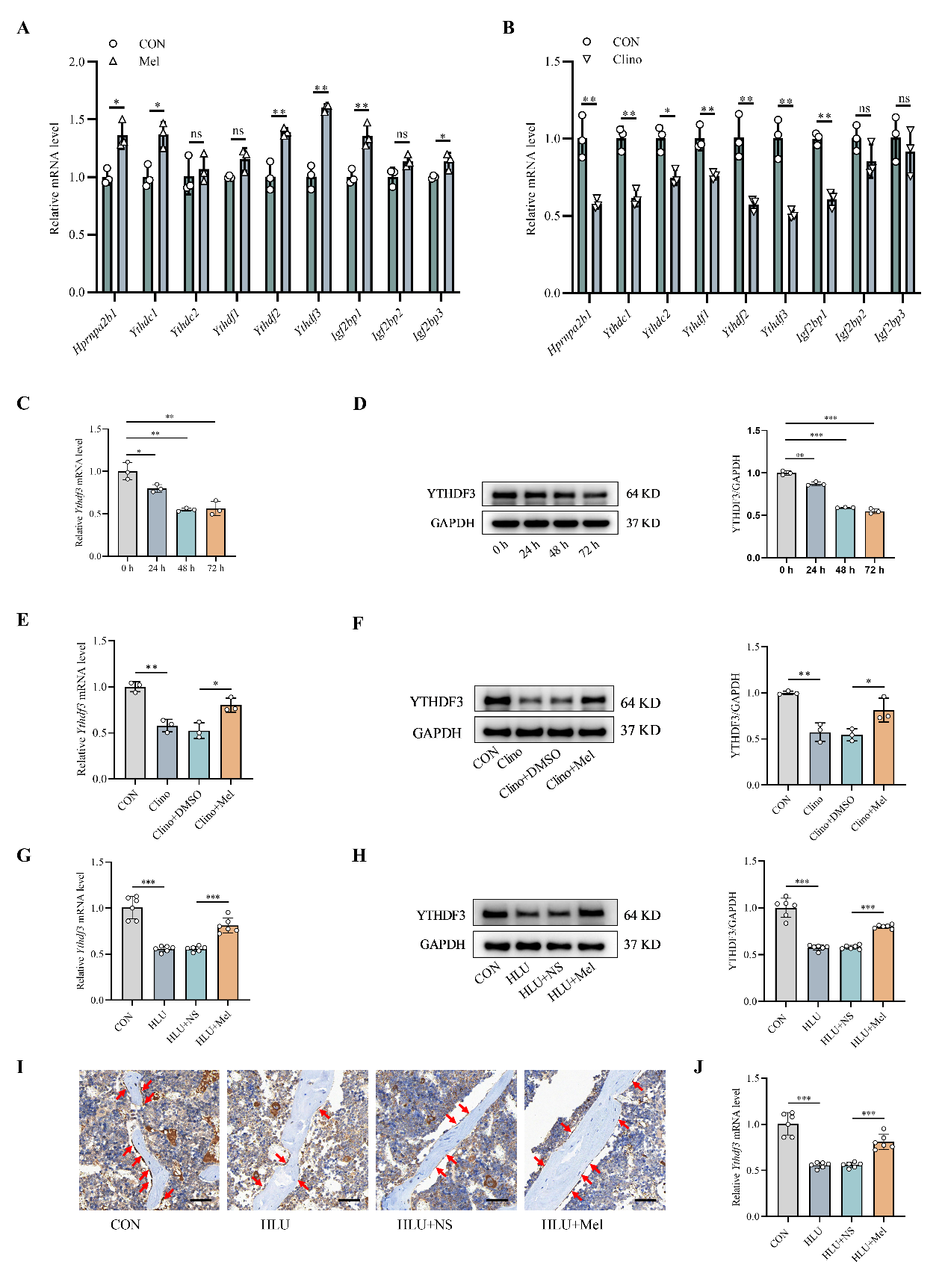
**

**Figure S3.** Melatonin effectively restores the expression of the mechanosensitive m^6^A reader YTHDF3, which is suppressed under mechanical unloading conditions. **(A)** Transcript levels of m^6^A reader proteins (*Hnrnpa2b1*, *Ythdc1*, *Ythdc2*, *Ythdf1*, *Ythdf2*, *Ythdf3*, *Igf2bp1*, *Igf2bp2*, and *Igf2bp3*) in MC3T3-E1 cells after melatonin exposure (n=3). **(B)** Expression profiles of m^6^A reader transcripts in MC3T3-E1 cells under mechanical unloading stimulation (n=3). **(C)** Temporal pattern of *Ythdf3* mRNA expression in MC3T3-E1 cells during 24–72 h of 2D clinorotation (n=3). **(D)** Time-dependent changes in YTHDF3 protein abundance in MC3T3-E1 cells under 2D clinorotation (24–72 h) (n=3). **(E)** Effect of melatonin on *Ythdf3* transcript levels in MC3T3-E1 cells subjected to 48 h of clinorotation (n=3). **(F)** Melatonin-mediated restoration of YTHDF3 protein expression in MC3T3-E1 cells under clinorotation (48 h) (n=3). **(G)** *Ythdf3* mRNA expression in the tibial bone of mice subjected to hindlimb unloading and melatonin supplementation (n=6). **(H)** YTHDF3 protein levels in the tibial bone of mice subjected to hindlimb unloading and melatonin treatment (n=6). **(I)** Representative IHC detection of YTHDF3 in distal femoral sections of HLU mice (n=6). Scale bar, 50 μm. **(J)** Quantitative assessment of YTHDF3-positive osteoblasts in the distal femoral regions of HLU mice (n=6). n=3 represents three independent biological replicates. ^*^*P* < 0.05, ^**^*P* < 0.01, ^***^*P* < 0.001 vs. control; ns=not significant.

**Figure S4**

**
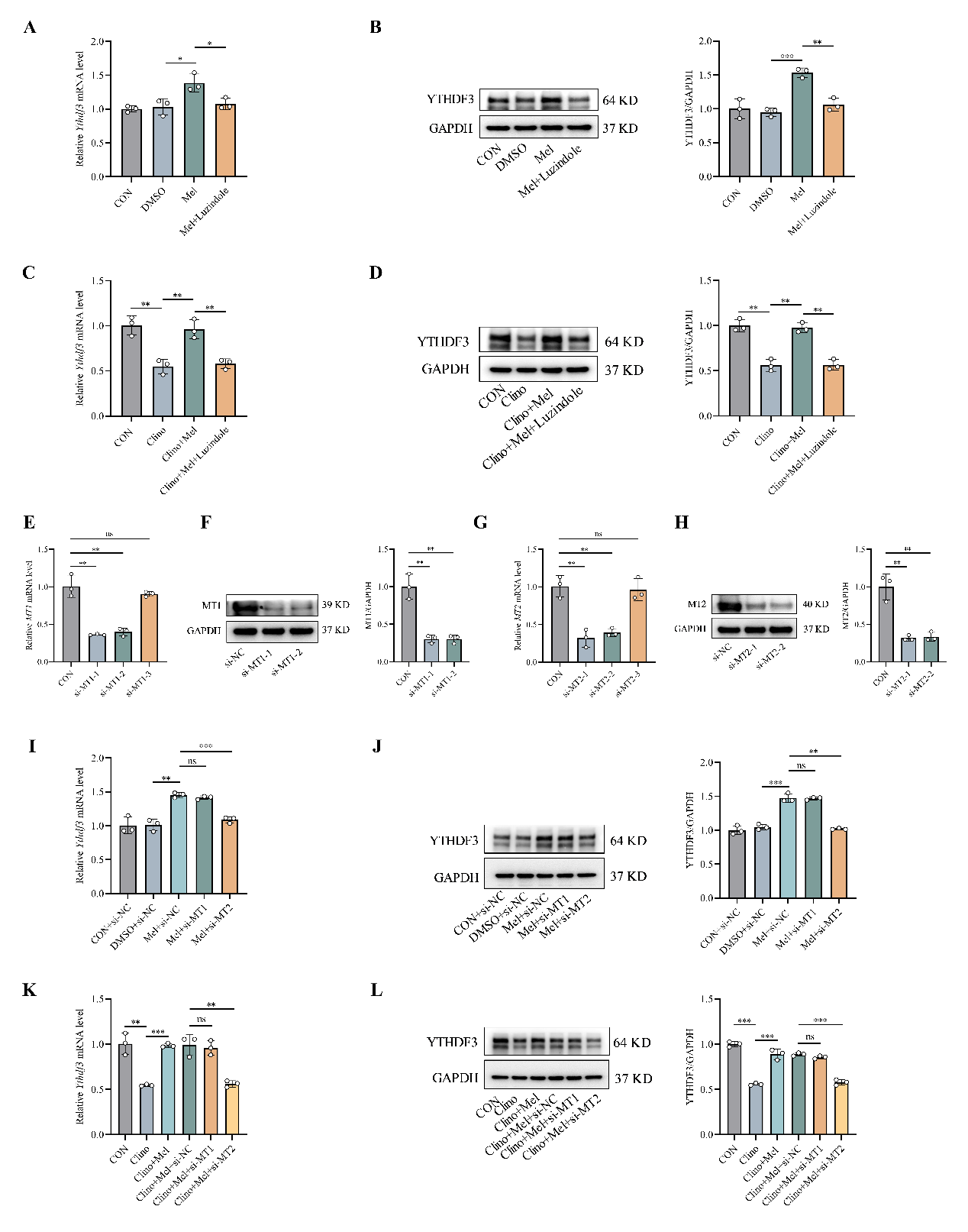
**

**Figure S4.** Melatonin Effectively Restores the Expression of the Mechanosensitive m6A Reader YTHDF3 through MT2 Receptor Under Mechanical Unloading Conditions. **(A)** mRNA expression levels of *Ythdf3* in MC3T3-E1 cells treated with Luzindole and melatonin for 48 hours (n=3). **(B)** Protein expression levels of YTHDF3 under the same treatment conditions as in (A) (n=3). **(C)** mRNA expression levels of *Ythdf3* in MC3T3-E1 cells treated with Luzindole and melatonin under 2D clinorotation for 48 hours (n=3). **(D)** Protein expression levels of YTHDF3 under the same treatment conditions as in (C) (n=3). **(E)** Validation of the efficacy of three independent siRNA sequences in suppressing MT1 mRNA expression (n=3). **(F)** Western blot confirmation of MT1 protein modulation through genetic knockdown approaches (n=3). **(G)** Validation of the efficacy of three independent siRNA sequences in suppressing MT2 mRNA expression (n=3). **(H)** Western blot confirmation of MT2 protein modulation through genetic knockdown approaches (n=3). **(I)** mRNA expression levels of *Ythdf3* in melatonin-treated MC3T3-E1 cells after transfection of si-MT1 or si-MT2 (n=3). **(J)** Protein expression levels of YTHDF3 under the same treatment conditions as in (I) (n=3). **(K)** mRNA expression levels of *Ythdf3* in melatonin-treated MC3T3-E1 cells after transfection of si-MT1 or si-MT2 under 2D clinorotation (n=3). **(L)** Protein expression levels of YTHDF3 under the same treatment conditions as in (K) (n=3). n=3 represents three independent biological replicates. ^*^*P* < 0.05, ^**^*P* < 0.01, ^***^*P* < 0.001 vs. control.

**Figure S5**


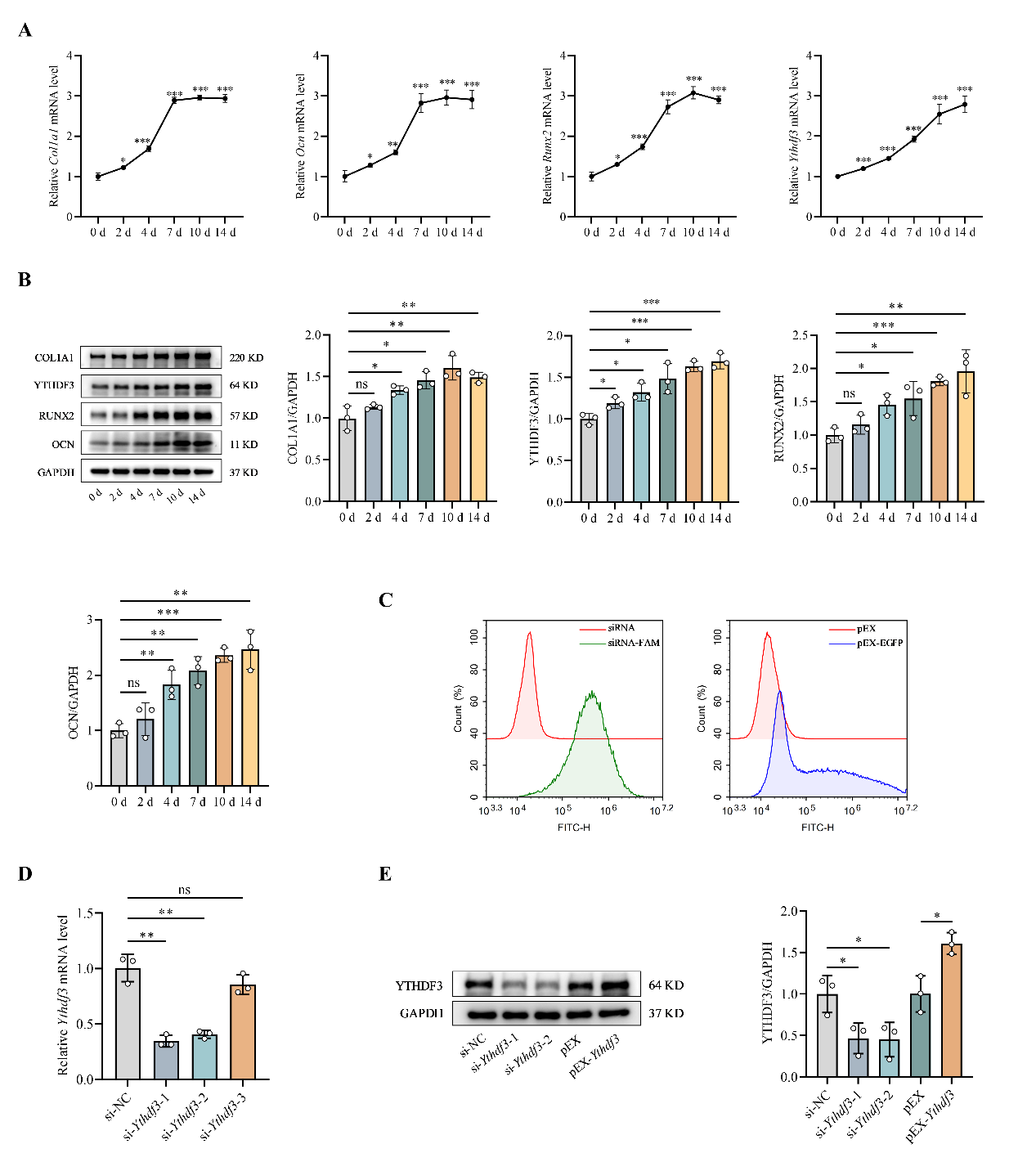


**Figure S5. (A)** Time course analysis of the transcriptional activation of osteogenic markers (*Col1a1*, *Ocn*, and *Runx2*) and *Ythdf3* during 2–14 days of osteogenic induction in MC3T3-E1 cells (n=3). **(B)** Parallel assessment of protein expression kinetics for osteogenic regulators (COL1A1, OCN, and RUNX2) and YTHDF3 throughout the differentiation timeline (n=3). **(C)** Quantitative evaluation of transfection efficiency via flow cytometry. **(D)** Validation of the efficacy of three independent siRNA sequences in suppressing *Ythdf3* mRNA expression (n=3). **(E)** Western blot confirmation of YTHDF3 protein modulation through genetic knockdown and overexpression approaches (n=3). n=3 represents three independent biological replicates. ^*^*P* < 0.05, ^**^*P* < 0.01, ^***^*P* < 0.001 vs. control; ns=not significant.

**Figure S6**

**
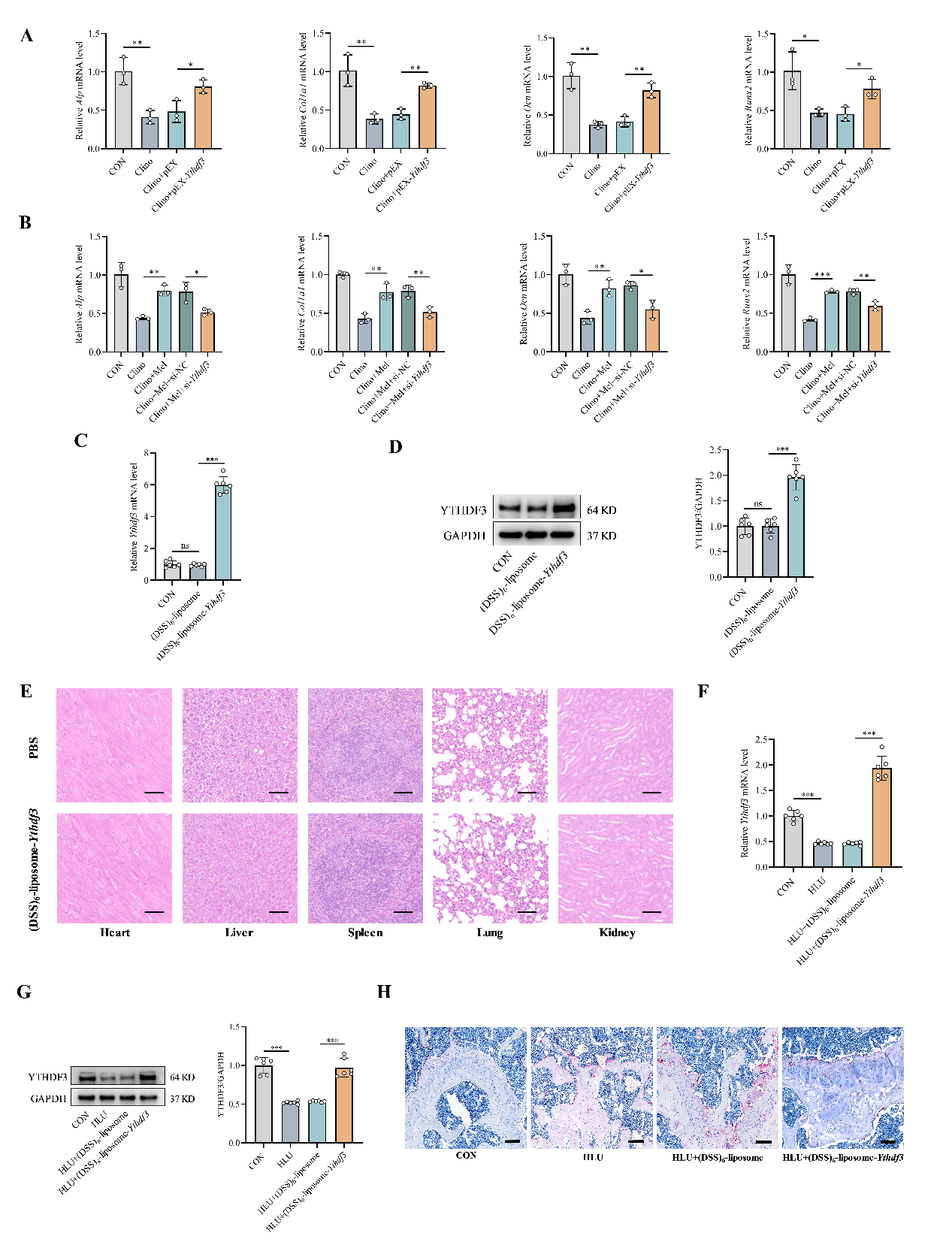
**

**Figure S6. (A)** Transcript levels of osteogenic differentiation markers in MC3T3-E1 cells overexpressing *Ythdf3* and exposed to 48 hours of clinorotation (n=3). **(B)** Quantitative analysis of osteogenic differentiation marker transcripts by qRT–PCR (n=3). **(****C)** *Ythdf3* mRNA levels in bone tissues after injection of (DSS)_6_-liposomes or (DSS)_6_-liposome-*Ythdf3* (n=6). **(D)** Western blot analysis of YTHDF3 protein levels in bone tissues after the injection of (DSS)_6_-liposomes or (DSS)_6_-liposome-*Ythdf3* (n=6). **(E)** Histopathological assessment by H&E staining demonstrating the tissue architecture of major organs (heart, liver, spleen, lung, and kidney) following treatment with either PBS or (DSS)_6_-liposome-*Ythdf3* (n=6). Scale bars, 100 μm. **(F)** mRNA expression of *Ythdf3* in the bone tissues of the hindlimbs in each group determined by qRT–PCR (n =6). **(G)** Protein expression of YTHDF3 in the bone tissues of the hindlimbs in each group determined by Western blot (n =6). **(H)** TRAP staining was used to examine osteoclast numbers and activity in murine tissue with femur bone. Scale bars, 100 μm. ^*^*P* < 0.05, ^**^*P* < 0.01, ^***^*P* < 0.001 vs. control; ns=not significant.

**Figure S7**

**
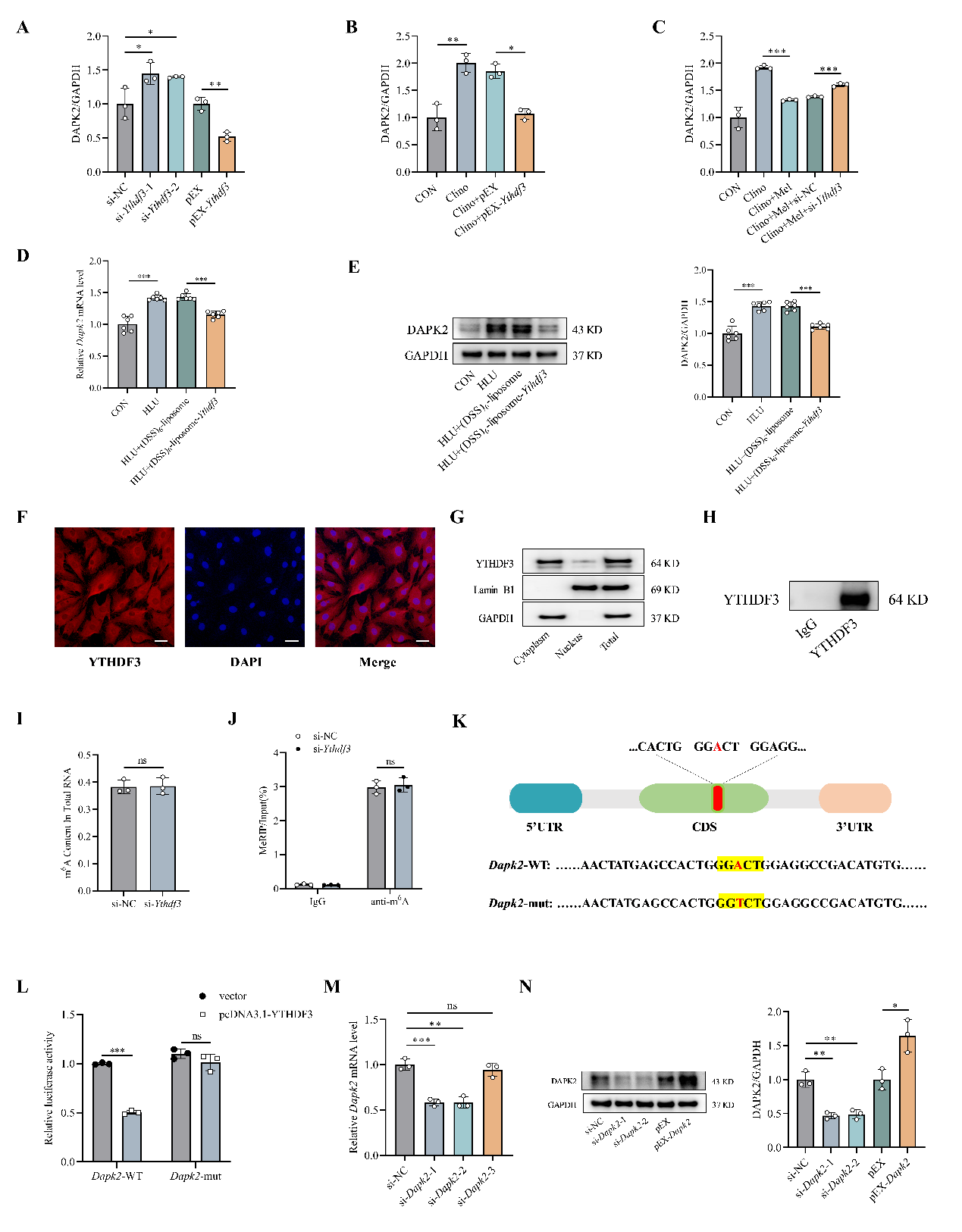
**

**Figure S7. (A)** Western blot quantification demonstrating DAPK2 protein regulation by genetic manipulation of YTHDF3 expression (n=3). **(B)** Statistical representation of DAPK2 protein modulation in YTHDF3-overexpressing osteoblasts under mechanical unloading conditions (n=3). **(C)** Quantitative Western blot analysis of DAPK2 expression in YTHDF3-silenced cells treated with melatonin during clinorotation (n=3). **(D)** Transcriptional profiling of *Dapk2* expression across experimental cohorts in murine models (n=6). **(E)** Comparative Western blot assessment of DAPK2 protein abundance in different treatment groups of mice (n=6). **(F)** Subcellular compartmentalization of YTHDF3 visualized by immunofluorescence microscopy. Scale bars, 50 μm. **(G)** Biochemical fractionation analysis of the cellular distribution of YTHDF3. **(H)** Validation of YTHDF3 antibody immunoreactivity for RIP studies. (**I**) Spectrophotometric quantification of global m^6^A methylation levels (n=3). **(J)** MeRIP-qPCR determination of site-specific m^6^A modifications on *Dapk2* transcripts following *Ythdf3* depletion (n=3). **(K)** Schematic representation of DAPK2-WT or DAPK2-mut sequence. **(L)** Dual-luciferase reporter assay measured the luciferase activities of DAPK2-WT or DAPK2-mut in 293T cells with YTHDF3 overexpression (n=3). **(M)** Validation of the efficacy of three independent siRNA sequences in suppressing *Dapk2* mRNA expression (n=3). **(N)** Western blot confirmation of DAPK2 protein modulation through genetic knockdown and overexpression approaches (n=3). n=3 represents three independent biological replicates. ^*^*P* < 0.05, ^**^*P* < 0.01, ^***^*P* < 0.001 vs. control; ns=not significant.

**Figure S8**


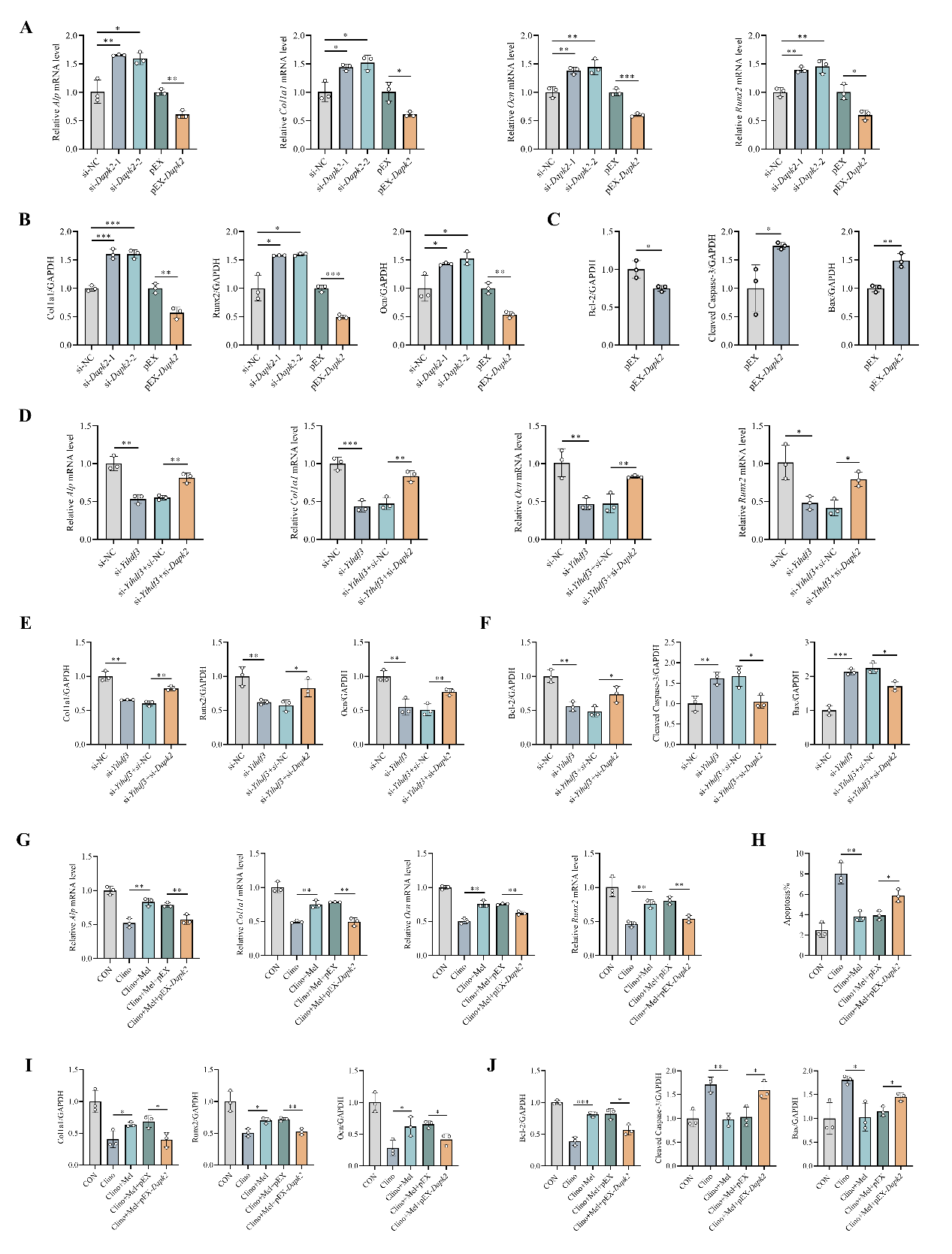


**Figure S8. (A)** Transcript levels of osteogenic markers quantified by qRT–PCR in MC3T3-E1 cells following *Dapk2* knockdown or overexpression (n=3). **(B)** Western blot analysis of key osteogenic differentiation proteins modulated by *Dapk2* manipulation (n=3). **(C)** Western blot analysis of apoptosis-related effectors, including proapoptotic proteins (Bax and cleaved caspase-3) and antiapoptotic Bcl-2 (n=3). **(D)** Quantitative assessment of osteogenic marker transcripts by qRT–PCR following dual transfection with si-*Ythdf3* and si-*Dapk2* in MC3T3-E1 cells (n=3). **(E)** Western blot analysis of key osteogenic differentiation regulators following dual transfection with si-*Ythdf3* and si-*Dapk2* in MC3T3-E1 cells (n=3). **(F)** Western blot evaluation of apoptosis-related proteins Bax, cleaved caspase-3, and Bcl-2 following dual transfection with si-*Ythdf3* and si-*Dapk2* in MC3T3-E1 cells (n=3). **(G)** Following transfection with pEX-*Dapk2*, MC3T3-E1 cells were maintained in melatonin-supplemented medium for 48 hours of clinorotation. Quantitative analysis of osteogenic differentiation marker transcripts by qRT–PCR (n=3). **(H)** Flow cytometric quantification of apoptosis (n=3). **(I)** Western blot evaluation of key osteogenic regulatory proteins (n=3). **(J)** Western blot assessment of the apoptosis-related proteins Bax, cleaved caspase-3, and Bcl-2 (n=3). n=3 represents three independent biological replicates. ^*^*P* < 0.05, ^**^*P* < 0.01, ^***^*P* < 0.001 vs. control.

**Table S1. Primers used for qRT**–**PCR**

| **Gene** | **Forward 5' - 3'** | **Reverse 5' - 3'** |
| --- | --- | --- |
| ***Gapdh*** | GCAAATTCAACGGCACAGTCAAG | TCGCTCCTGGAAGATGGTGATG |
| ***Runx2*** | GAACCAAGAAGGCACAGACAGA | GGCGGGACACCTACTCTCATAC |
| ***Alp*** | AACTGATGTGGAATACGAACTGGATG | CATAGTGGGAATGCTTGTGTCTGG |
| ***Bglap*** | AGCAGGAGGGCAATAAGGTAGTG | CTCGTCACAAGCAGGGTTAAGC |
| ***Col1a1*** | AGGCGAACAAGGTGACAGAGG | GGAGAACCAGGAGAACCAGGAG |
| ***Hnrnpa2b1*** | GCGGAGGAAGAGGCGGTTAC | GTTAGAAGGCTGCTGGTTGTAGTTG |
| ***Igf2bp1*** | CCTGGCTCATAACAACTTCGTC | TGATCTTCGTCTCTGTGTCCTG |
| ***Igf2bp2*** | TCCAGCAGAAGGTCCAGATGTC | TTCCCAAAGATCCGTCCCTGAG |
| ***Igf2bp3*** | TCCAGCCGAAGCACCAGATG | TCTTCCCTGAGCCTTGAACTGAG |
| ***Ythdc1*** | TCAGGAGTTCGCCGAGATGTG | GTAAGGATGGTGTGGAGGTTGTTC |
| ***Ythdc2*** | CCTGTTACTGTCCTGGTGTTCTG | ATCTCACTGTCACTGCTGTCATTG |
| ***Ythdf1*** | GTCCAGTTACTACTATCCACCATCCATTG | AGTTGTCCATAGGTAGTGAGATACGG |
| ***Ythdf2*** | TTGCCTCCACCTCCACCACAG | CCCATTATGACCGAACCCACTGC |
| ***Ythdf3*** | AGCCTTGAGCAGCAGTGGTATG | ACATTGCCCTTGGGTTTAAGTTTCG |
| ***Dapk2*** | GGAGCATTGGAGTCATCACCTATATC | TTCCTCATCAAAGTCGTAACTCACAG |
| ***Acta1*** | ACCATCGGCAATGAGCGTTTC | GTTGTTGGCATACAGGTCCTTCC |
| ***Cdh4*** | GGACAGTCTTCGCCACTAGGG | ACCAGCAGCCGCACCATAG |
| ***Cyp1a1*** | AGAAGGTGATGGCAGAGGTTGG | ATATGGCACAGATGACATTGGCTAC |
| ***Gpc3*** | TCAACACTACCGACCACCTCAAG | AATAACCACCGCAAGGCTTAACC |
| ***Sdk2*** | CACAATGCCTCCGCCTTCAC | GATGTCGTTGGTCGCCTTCAC |
| ***Zfp541*** | CAGAGTCAGAGTTGGAGTCAGAGTC | GCAGGCAGGCAGGCAGAG |
| ***Tspan3*** | GCGGCATCACCTCCTCTAGAC | CGGCAGGGAAGAGCGTGTAC |
| ***Sla2*** | TGAGGATGGAGATTGGTGGACAG | ACCCGTGGGCGACTTTAGC |
| ***MT1*** | AGCTCAGCGTACACGATAGC | GCTTGTTGTCGGGTTTCACC |
| ***MT2*** | CTCGTCTGGCTCCTCACTCTG | TGCTGGCTGTCTGGATGAAGG |

**Table S2. siRNAs sequences**

| **Name** | **Sequence 5' - 3'** |
| --- | --- |
| **si-*Ythdf3*-1 sense** | GCAGUGGUAUGACHAGCAUTT |
| **si-*Ythdf3*-1 antisense** | AUGCUAGUCAUACCACUGCTT |
| **si-*Ythdf3*-2 sense** | CCACCACCAUUGGUGCAAATT |
| **si-*Ythdf3*-2 antisense** | UUUGCACCAAUGGUGGUGGTT |
| **si-*Dapk2*-1 sense** | CAGUUUGCCAUCGUGAAGATT |
| **si-*Dapk2*-1 antisense** | UCUUCACGAUGGCAAACUGTT |
| **si-*Dapk2*-2 sense** | GAGAACUGUUUGAUUUCCUTT |
| **si-*Dapk2*-2 antisense** | AGGAAAUCAAACAGUUCUCTT |
| **si-*MT1*-1 sense** | GGUGGCUGUUUACCCUUAUTT |
| **si-MT1-1 antisense** | AUAAGGGUAAACAGCCACCTT |
| **si-*MT1*-2 sense** | GGCUCGAUAUUCAACAUCATT |
| **si-*MT1*-2 antisense** | UGAUGUUGAAUAUCGAGCCTT |
| **si-*MT2*-1 sense** | CUCCCAUGCUAUCUACAGUTT |
| **si-*MT2*-1 antisense** | ACUGUAGAUAGCAUGGGAGTT |
| **si-*MT2*-2 sense** | GAUCCACGCAUCUAUUCCUTT |
| **si-*MT2*-2 antisense** | AGGAAUAGAUGCGUGGAUCTT |
| **si-*Dapk2*-2 antisense** | AGGAAAUCAAACAGUUCUCTT |
| **si-NC antisense** | ACGUGACACGUUCGGAGAATT |
